# Prognostic value of antitumor drug targets prediction using integrated bioinformatic analysis for immunogenic cell death-related lncRNA model based on stomach adenocarcinoma characteristics and tumor immune microenvironment

**DOI:** 10.3389/fphar.2022.1022294

**Published:** 2022-10-14

**Authors:** Dayong Ding, Yan Zhao, Yanzhuo Su, Huaixi Yang, Xuefeng Wang, Lin Chen

**Affiliations:** ^1^ Department of Gastrointestinal and Colorectal Surgery, China-Japan Union Hospital of Jilin University, Changchun, Jilin, China; ^2^ Department of Operating Room, China-Japan Union Hospital of Jilin University, Changchun, Jilin, China

**Keywords:** stomach adenocarcinoma, ICD, lncRNA, prognostic, TCGA, tumor-infiltrating immune cells

## Abstract

Stomach adenocarcinoma (STAD) ranks as the fourth prevalent cause of mortality worldwide due to cancer. The prognosis for those suffering from STAD was bleak. Immunogenic cell death (ICD), a form of induced cellular death that causes an adaptive immune response and has increasing in anticancer treatment. However, it has not been ascertained how ICD-related lncRNAs affect STAD. Using univariate Cox regression and the TCGA database, lncRNAs with prognostic value were identified. Thereafter, we created a prognostic lncRNA-based model using LASSO. Kaplan-Meier assessment, time-dependent receiver operating characteristic (ROC) analyzation, independent prognostic investigation, and nomogram were used to assess model correctness. Additional research included evaluations of the immunological microenvironment, gene set enrichment analyses (GSEA), tumor mutation burdens (TMBs), tumor immune dysfunctions and exclusions (TIDEs), and antitumor compounds IC50 predictions. We found 24 ICD-related lncRNAs with prognostic value *via* univariate Cox analysis (*p* < 0.05). Subsequently, a risk model was proposed using five lncRNAs relevant to ICD. The risk signature, correlated with immune cell infiltration, had strong predictive performance. Individuals at low-risk group outlived those at high risk (*p* < 0.001). An evaluation of the 5-lncRNA risk mode including ROC curves, nomograms, and correction curves confirmed its predictive capability. The findings of functional tests revealed a substantial alteration in immunological conditions and the IC50 sensitivity for the two groups. Using five ICD-related lncRNAs, the authors developed a new risk model for STAD patients that could predict their cumulative overall survival rate and guide their individual treatment.

## Introduction

Stomach cancer is fifth in terms of prevalence and fourth in regards of fatality leading to over one million new cases in 2020 (Sung et al., 2021). Stomach adenocarcinoma (STAD) was the most common and malignant subtype. Surgical treatment is the primary method of treating STAD. Patients are typically identified at an advanced phase, with a dismal prognosis. Patients with advanced STAD typically have a 5-year survival rate fewer than 5% (Meng et al., 2021). Molecular mechanisms underlying STAD still have not been fully clarified. Consequently, further research into new indicators that affect STAD prognosis is required, by categorizing patients with different prognoses, and improving prognosis prediction, which is significant to improve early diagnosis and treatment of STAD.

Immunogenic cell death (ICD) is a type of cancer cell death that can boost the activation of the immune system against tumor in immunocompetent hosts. ICD comprises the release of damage-associated molecular patterns (DAMPs), such as the cell surface exposure of calreticulin (CRT), heat-shock proteins (HSP70 and HSP90), extracellular ATP, high-mobility group box-1 (HMGB1), and type I IFNs etc., from dying tumor cells that result in the activation of tumor-specific immune responses (Zhou et al., 2019; Wang et al., 2021a). ICD can be triggered by certain chemotherapeutic drugs, oncolytic viruses, photodynamic therapy, and radiotherapy. Crizotinib, an ICD-inducing tyrosine kinase inhibitor, exhibits exceptional antineoplastic effect when coupled of non-ICD-inducing chemotherapy drugs like cisplatin. Chemotherapy causes the overexpression of immunosuppressive molecule in gastric cancer: programmed death-ligand 1 (PD-L1) (Petersen et al., 2021). Immune responses to cancer are stimulated by ICD when dying cancer cells are converted into therapeutic vaccines. Tumors with a high propensity for ICD may elicit a more vigorous immune response, aiding in the fight against and slowing down tumor growth. Therefore, further clinical studies are required to determine the possibilities of ICD-related immunotherapy.

Long noncoding RNAs (lncRNAs) have more than 200 nucleotides and regulate various biological processes in cancers (Wang et al., 2021b). NcRNAs affect gastric cancer chemo- and immunotherapy resistance. EIF3J-DT induces STAD chemoresistance by stimulating autophagy (Luo et al., 2021); MEG3 inhibits STAD proliferation and metastasis by blocking p53 signaling (Wei and Wang, 2017). Oncogenic lncRNAs such as plasmacytoma variant translocation 1 (PVT1) have been linked to chemoresistance (Wei et al., 2020). Meanwhile, several lncRNAs, including ferroptosis-related lncRNAs (Wei et al., 2021; Wang et al., 2022), immune-related lncRNAs (Zhi et al., 2022), and m5C-related lncRNAs (He et al., 2022), have recently been linked to STAD prognosis. The impact of ICD-associated lncRNAs on STAD, however, remains unclear.

Herein, we established a risk model based on five ICD-associated lncRNAs to predict STAD patients’ prognosis, immunological microenvironment, and chemosensitivity. In line with our expectations, our model showed a good prediction of survival ability in STAD patients and depicted the characteristics of immune cell invasion, immunological checkpoints, and drug sensitivity, providing individualized treatment for STAD patients.

## Materials and methods

### Data preprocessing

375 cancer samples and 32 non-cancerous samples of TPM RNA-seq data were obtained from the TCGA database (https://tcga-data.nci.nih.gov/tcga/). The clinical information, such as age, gender, and survival information, were retrieved. Perl programming language was used to combine these data into a matrix file. Patients that clinical information cannot access were deleted from subsequent analyses. Next, 34 ICD-related genes were retrieved from published research (Garg et al., 2015).

### Examination of differentially expressed ICD-related lncRNAs

Using the R package “limma” in combination with the specifications: Pearson |coefficient correlation| > 0.40 and *p* < 0.001, 572 ICD-associated lncRNAs were identified as ICD-associated lncRNAs. Based on these lncRNAs, adjust *p* < 0.05 thresholds for lncRNAs with |log_2_ (fold change, FC)| > 1 between STAD samples and non-carcinoma samples were screened out as differentially expressed ICD-related lncRNAs.

### Construction of prognostic model for ICD-related lncRNAs

Survival-related lncRNAs from differentially expressed ICD-related lncRNAs were conducted using univariate Cox regression analysis with STAD patients’ clinical data. The prognostic lncRNAs were then subjected to reduce overfitting with Lasso-Cox regression method. Then, a risk model with a *p*-value less than 0.05 and one thousand cycles was constructed. The risk scores were calculated according to this formula:

$$risk\;score=\overset{n}{\underset{i=1}{\sum }}\left(coef_{i}*\exp r_{i}\right)$$ (1)

The $coef_{i}$ represents each lncRNA’s coefficient index, and $\exp r_{i}$ represents each lncRNA’s expression level.

### Verification of the risk model

The Kaplan–Meier (KM) analysis of survival validated our ICD-related lncRNA risk model. First, entire TCGA cohort was discretized into training and validation groups at a 1:1 ratio. Observations were randomly assigned into low-risk or high-risk groups based on median risk scores. Chi-square test was utilized to evaluate the risk model’s predictive efficacy. Using the packages “survival” and “survminer”, survival curves were obtained based on the number of events in each group. Using the validation set, the dependability of risk scores was evaluated. With STAD patients’ clinical data, we established univariate and multivariate Cox regressions. Data on age, gender, grade, and stage were collected to determine the independence of the risk score as a predictor of overall survival (OS). The prognostic effectiveness was evaluated using Harrell’s concordance index (C-index) and time-dependent ROC curve using R packages “survcomp” and “survivalROC”.

### Nomogram establishment

To determine the consistency of the prediction outcome, The nomogram was established utilizing risk score, age, gender, and tumor stage. The “rms” R package software was used to generate line graphs for the 1-, 2-, and 3-year OS with Hosmer-Lemeshow correction curves.

### Immune cell infiltration and checkpoints analysis

CIBERSORT is used for deconvoluting immune cell expression matrices using linear support vector regression, it counts the percentage of immune cells in infiltrate tissues with the detection of marker gene expression. In this study, we used the CIBERSORT method with the leukocyte gene signature matrix (LM22) as a reference and 1,000 permutations to measure immune cells ratio in tumor microenvironment (TME). The “ESTIMATE” R program was used to determine the stromal cell and immune cell scores, which are subsequently used to assess the tumor purity. In addition, immune cell invasion, functional pathways, immune cell correlations with risk scores, and immunological checkpoints were examined in comparisons between the risk groups with “ggpubr” R package. The correlation between the risk score and infiltrating immunocytes was examined using Spearman’s test. A *p*-value of less than 0.05 was deemed significant.

### Gene set enrichment analyses enrichment analysis

We examined the alteration in gene expression between different risk cohorts with the MSigDB enrichment dataset (c2.cp.kegg.v7.4.symbols.gmt) using the R package “GSEA”.

### Tumor immune dysfunctions and exclusions analysis

The Tumor Immune Dysfunction and Exclusion (TIDE) approach can assess T cells malfunction, exclusion, and checkpoint inhibitors response (http://tide.dfci.harvard.edu/) (Jiang et al., 2018). TIDE scores that are higher indicate a greater likelihood of antitumor immune escape.

### Tumor mutation burden analysis

The somatic mutation profile was obtained and R package “maftools” was used to examine Mutation Annotation Format (MAF) data on somatic mutations. For each STAD instance, a tumor mutation burden (TMB) score was calculated using the methodology below:

$$TMB=\frac{total\;mutation}{total\;covered\;bases}\times 10^{6}$$ (2)

### Gene ontology and kyoto encyclopedia of genes and genomes enrichment

We identified signal pathways and biological effects using the Kyoto Encyclopedia of Genes and Genomes (KEGG) and Gene Ontology (GO) analysis. The *p*-value and *q*-value criteria of 0.05 thresholds were employed with the “clusterProfiler” package, FDR and *p* < 0.05 were regarded as significantly enriched.

### Analysis of clinical chemotherapy risk model performance

With the “pRRophetic” package, the half-maximal inhibitory concentrations (IC50) of anti-cancer medicines were administered to a variety of subgroups of patients to assess how effectively a substance inhibits a biological process, *p* values <0.001 were considered statistically significant.

### Statistical analysis

All statistical analyses were conducted using R, version 4.1.3. Using Pearson Correlation examination, the relationships between ICD-related genes and lncRNAs were examined. Immune infiltrating cells were analyzed using Wilcoxon signed-rank test. A *p*-value below 0.05 indicated statistical significance.

## Results

### Identification of differentially expressed ICD-related lncRNAs

**FIGURE 1:**
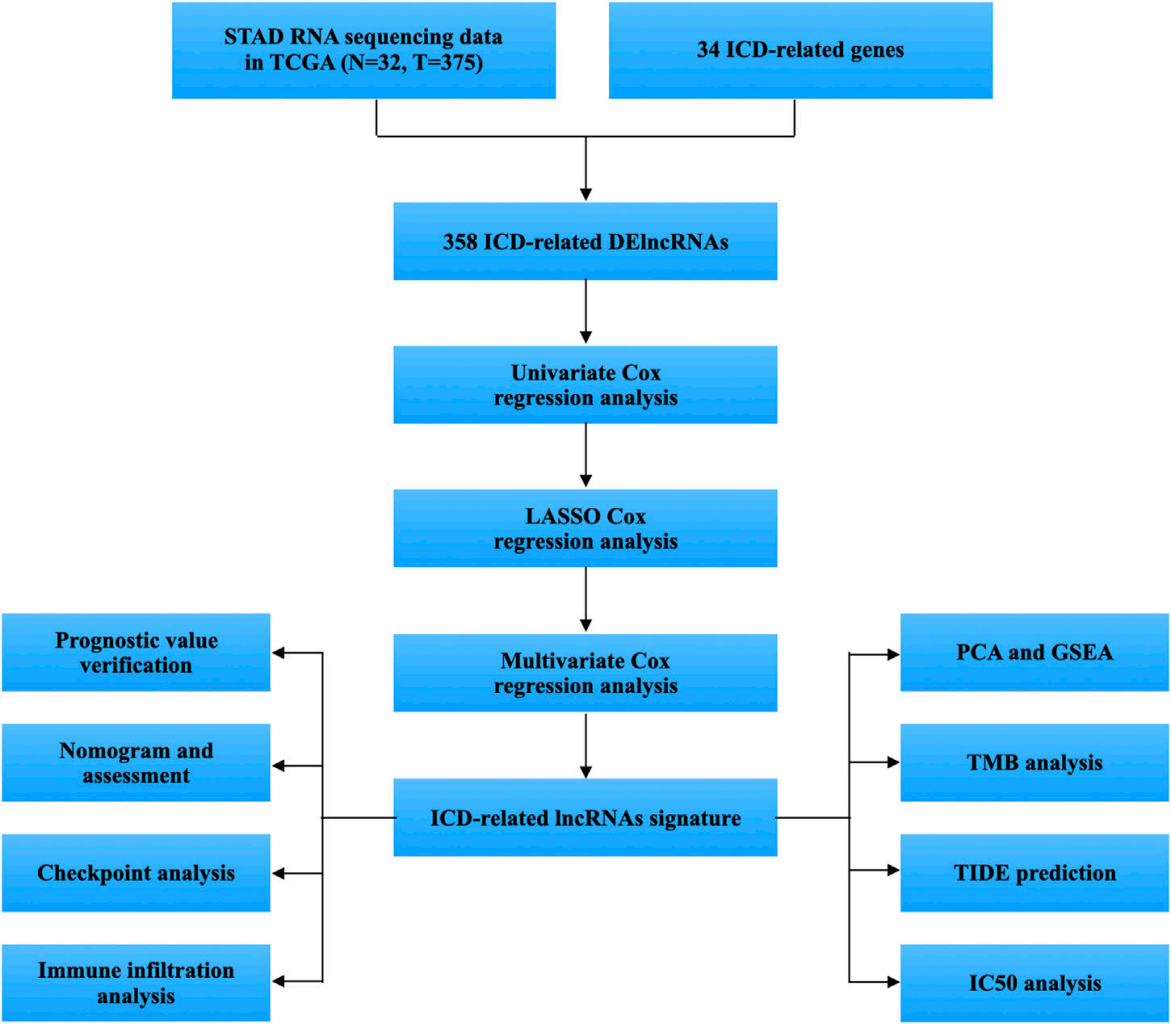
Workflow of the study.

**FIGURE 2:**
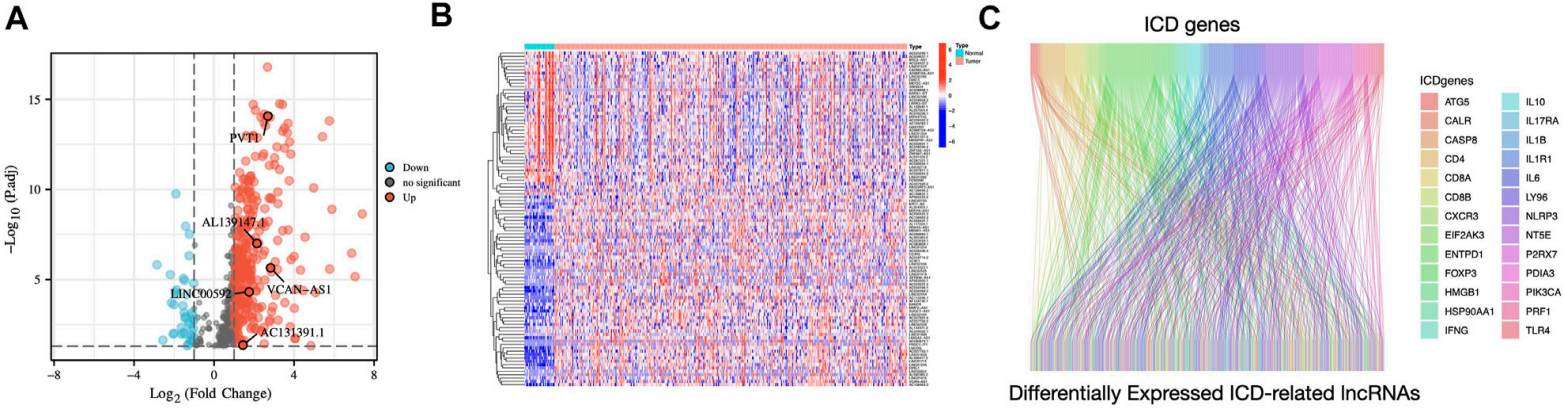
Identification of differentially expressed ICD-related lncRNAs in STAD patients **(A)** Volcano plot illustrating the differential expression lncRNAs in STAD samples compared to those in non-carcinoma tissues **(B)** Heatmap of ICD-related differentially expressed lncRNAs **(C)** The Sankey diagram illustrates the correlation between ICD genes and ICD-related lncRNAs.

Figure 1 displays the study’s flow diagram. 572 ICD-related lncRNAs were determined from STAD samples. Of these, 358 lncRNAs were differentially expressed ICD-related lncRNAs (Figures 2A,B). Co-expression networks of 34 ICD-related genes and differently expressed lncRNAs are shown in Figure 2C.

### Establishing a 5 ICD-related lncRNA risk model for STAD patients

**FIGURE 3:**
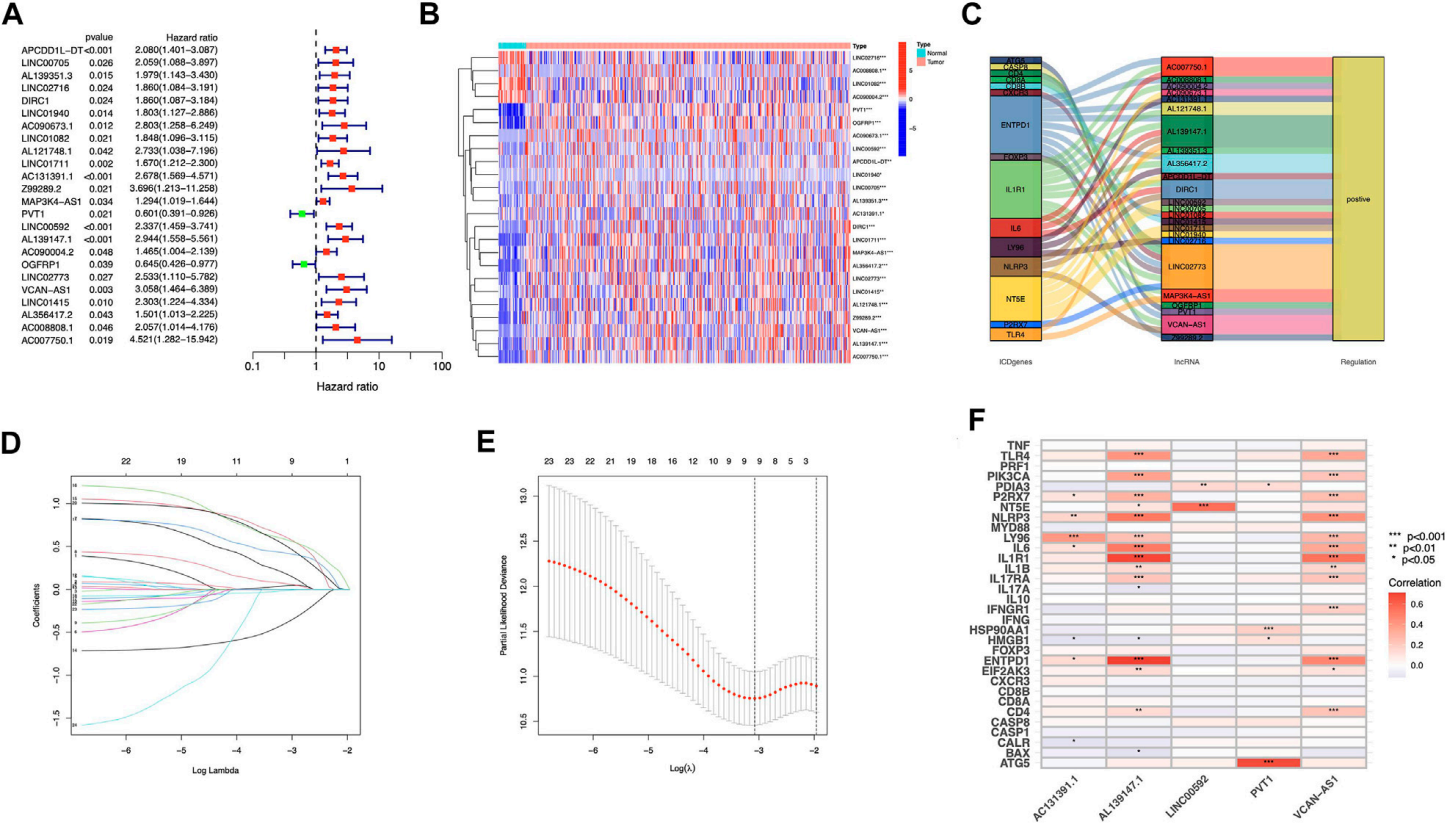
Construction of prognostic model for ICD-related lncRNAs in STAD patients **(A)** Analysis of 24 lncRNAs associated with prognosis using univariate Cox regression (*p* < 0.05) **(B)** 24 lncRNAs associated to prognosis were expressed differentially in adenocarcinomas and healthy stomach tissues. **(C)** The correlations between prognosis-related lncRNAs and corresponding ICD genes **(D)** Distribution of LASSO coefficients for 5 ICD lncRNAs. **(E)** A cross-validation procedure for optimizing LASSO regression parameters **(F)** The correlations between the five prognosis-related lncRNAs and ICD-related genes.

**FIGURE 4:**
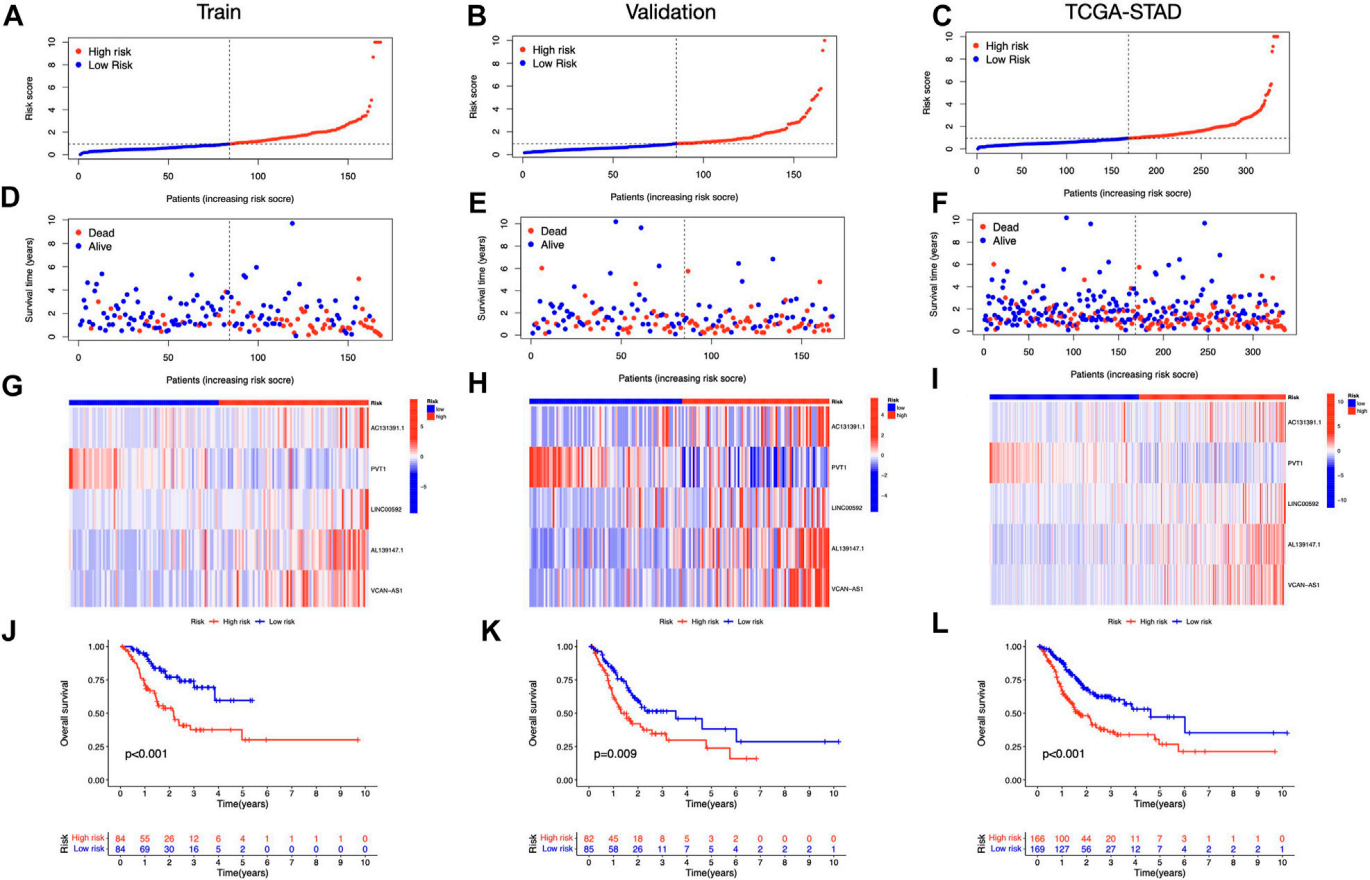
Prognosis value of the five ICD-related lncRNAs model **(A–C)** Illustration of an ICD-related lncRNA model depending on risk scores for train, validation, and entire sets, respectively. **(D–F)** Low- and high-risk groups’ train, validation, and whole-set survival times and statuses are compared **(G–I)** Heat maps depict 5 lncRNAs in train, validation, and entire datasets **(J–L)** OS curves for low- and high-risk groups in train, validation, and entire sets.

We discovered 24 prognostic ICD-related lncRNAs using univariate Cox regression (*p* < 0.05) (Figure 3A), based on which a heatmap was drafted (Figure 3B). Further, these lncRNAs are positively regulated by their associated genes (Figure 3C). The Lasso regression was performed, and five lncRNAs were sorted out (Figures 3D,E). Besides, the correlation between the five lncRNAs and ICD genes were shown in Figure 3F. The risk score for each sample was computed using the approach given below: Risk Score = AC131391.1 × (0.6469) + PVT1 × (-0.8239) + LINC00592 × (1.0268) + AL139147.1 × (0.8928) + VCAN-AS1 × (0.9535). Using the algorithm above, a median score was determined, and the TCGA-STAD cohort, training, and validation entities were partitioned into low- and high-risk cohorts. As shown in Figure 4, a five ICD-related lncRNA prognostic model was established to predict patients’ OS. The risk score distribution is shown in Figures 4A–C. In the high-risk category, there were more deceased observations (Figures 4D–F). The expression of the five risk lncRNAs in each group was shown in Figures 4G–I. Observations in low-risk group outlived those from the high-risk group, according to KM plots (Figures 4J-L). The detailed clinical parameters for training and test set were shown in Table 1.

**TABLE 1:** Clinicopathologic characteristics of STAD patients.

Features	Total	Test	Train	*p*-value
Age				0.5879
<=65	153(45.67%)	79(47.31%)	74(44.05%)	
>65	179(53.43%)	86(51.5%)	93(55.36%)	
unknown	3(0.9%)	2(1.2%)	1(0.6%)	
Gender				0.2233
FEMALE	118(35.22%)	53(31.74%)	65(38.69%)	
MALE	217(64.78%)	114(68.26%)	103(61.31%)	
Grade				0.2657
G1	9(2.69%)	6(3.59%)	3(1.79%)	
G2	120(35.82%)	53(31.74%)	67(39.88%)	
G3	197(58.81%)	101(60.48%)	96(57.14%)	
unknown	9(2.69%)	7(4.19%)	2(1.19%)	
Stage				0.8702
Stage I	44(13.13%)	19(11.38%)	25(14.88%)	
Stage II	107(31.94%)	54(32.34%)	53(31.55%)	
Stage III	137(40.9%)	68(40.72%)	69(41.07%)	
Stage IV	33(9.85%)	16(9.58%)	17(10.12%)	
unknown	14(4.18%)	10(5.99%)	4(2.38%)	
T				0.1643
T1	15(4.48%)	5(2.99%)	10(5.95%)	
T2	73(21.79%)	38(22.75%)	35(20.83%)	
T3	155(46.27%)	71(42.51%)	84(50%)	
T4	88(26.27%)	51(30.54%)	37(22.02%)	
unknown	4(1.19%)	2(1.2%)	2(1.19%)	
M				1
M0	302(90.15%)	149(89.22%)	153(91.07%)	
M1	21(6.27%)	10(5.99%)	11(6.55%)	
unknown	12(3.58%)	8(4.79%)	4(2.38%)	
N				0.222
N0	98(29.25%)	42(25.15%)	56(33.33%)	
N1	91(27.16%)	52(31.14%)	39(23.21%)	
N2	67(20%)	30(17.96%)	37(22.02%)	
N3	68(20.3%)	34(20.36%)	34(20.24%)	
unknown	11(3.28%)	9(5.39%)	2(1.19%)	

### Independent prognostic analysis

**FIGURE 5:**
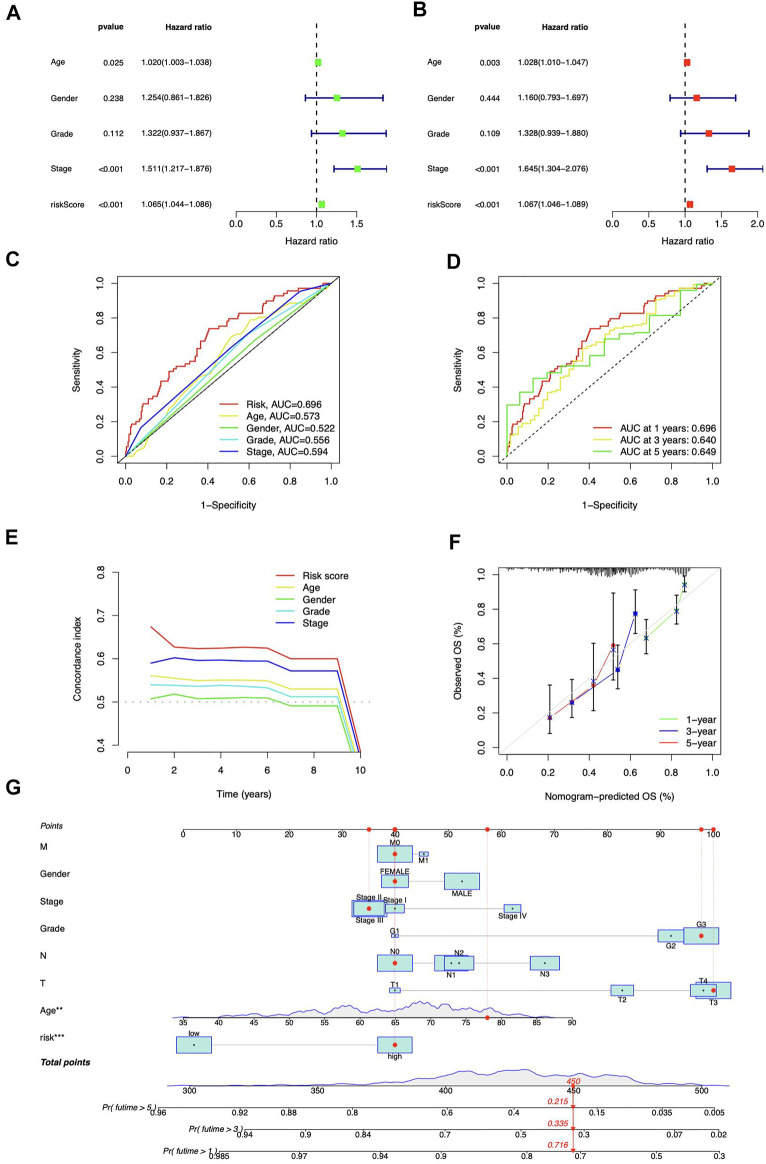
Prognostic analysis of ICD-related lncRNAs. Univariate **(A)** and multivariate **(B)** Cox regression for clinical features and risk model prognosis **(C)** Predictive accuracy of clinical characteristics and the risk model **(D)** ROC curve with a time dependence indicating the OS rates at 1, 3, and 5 years **(E)** C-index scores of clinical features and the risk model **(F)** Calibration curves for 1-, 3-, and 5-year survival predictions **(G)** The nomogram integrated the risk score, age, grade, T, M, N, and tumor stage to predict OS.

Both risk score and stage demonstrated substantial connections with the patients’ prognosis, as shown by a univariate Cox regression analysis (Figure 5A). Utilizing multivariate analysis, the risk score and stage independently predicted STAD patients’ OS (Figure 5B). The risk signature was more predictive than other clinical indications, according to ROC curves (Figure 5C). At 1, 3, and 5 years, the area under the curve (AUC) reaches 0.696, 0.640, and 0.649, respectively (Figure 5D). The risk signature also scored highest from C-index curves (Figure 5E). According to our findings, the ICD-related lncRNAs risk model exhibited stronger prognostic prediction for STAD than classical clinical and pathological features.

### Construction of prognostic nomogram

The nomogram was developed (Figure 5G) to enhance the survival prediction for STAD patients. The 1-, 3-, and 5-year OS calibration curves demonstrated high agreement between predictions and observations (Figure 5F).

### Relationship between immunological traits and risk model

**FIGURE 6:**
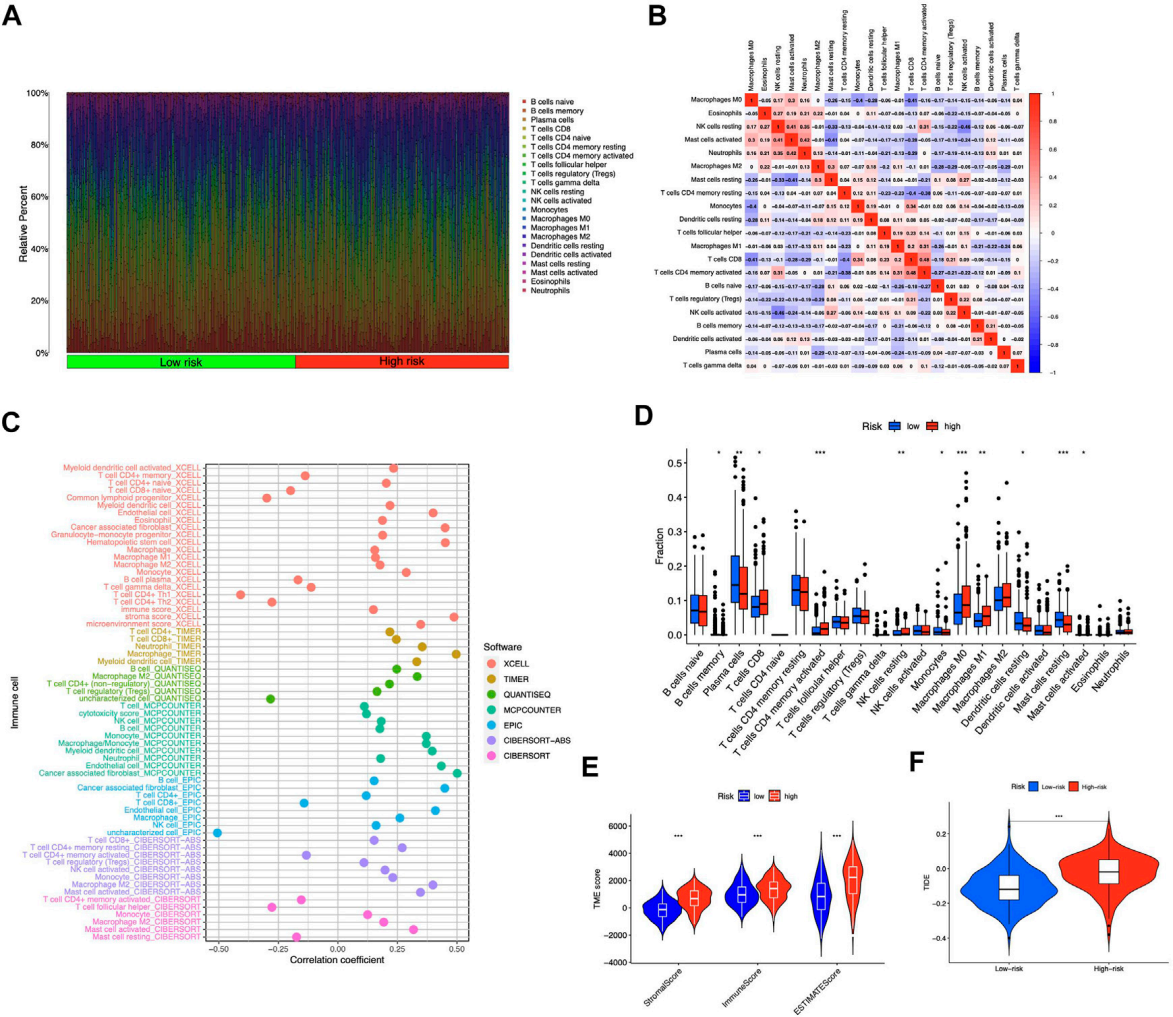
Tumor-infiltrating lymphocytes in the risk model **(A)** The violet plot showed the distribution of 22 tumor-infiltrating lymph cells of STAD patients in high- and low-risk groups **(B)** The correlation of the immune cells **(C)** Immune cell and risk score correlation **(D)** Comparing immune cells from high- and low-risk individuals **(E)** Comparison of ESTIMATE scores between groups with high and low risk **(F)** Comparison of TIDE scores between groups with high and low risk. *p < 0.05, **p < 0.01, ***p < 0.001 vs. low-risk group.

**FIGURE 7:**
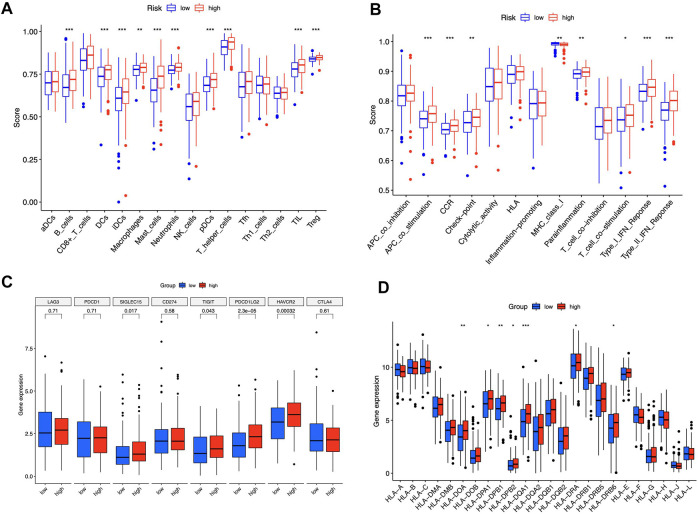
Immune signature of the risk model. ssGSEA enrichment of immune cells **(A)**. Immune function **(B)**. Box plots illustrated different expressions of immune checkpoints **(C)** and HLA genes **(D)** in groups with high and low risk. *p < 0.05, **p < 0.01, ***p < 0.001 vs. low-risk group.

We contrasted the variations in the tumor microenvironment between the groups with high- or low-risk. 22 immune cells that infiltrated tumors were examined (Figure 6A), and a significant correlation between immune cells was observed (Figure 6B). The bubble diagram showed the correlations between immune cells and risk scores (Figure 6C). Most immune cells showed a positive correlation with risk scores, especially hematopoietic stem cells of XCELL, resting memory CD4^+^ T cells, activated mast cells, M2 macrophages of CIBERSORT-ABS, B cells of QUANTISEQ, and cancer-associated fibroblasts of MCPCOUNTER. Additionally, memory B cells, resting dendritic cells, plasma cells, monocytes, and resting mast cells were more abundant in the low-risk group in comparison to the high-risk group (*p* < 0.05), whereas the high-risk group had greater levels of expression of activated CD4^+^ memory T cells, resting NK cells, and M0 macrophages (Figure 6D). Furthermore, the tumor microenvironment score was determined by “ESTIMATE” to quantify stromal-immune cell proportions. High-risk STAD individuals had higher stromal, immunological, and overall ESTIMATE scores (Figure 6E), demonstrating a greater immunological fraction in the high-risk group. Then, those in high-risk category exhibited higher TIDE scores (Figure 6F), suggesting that patients with high-risk ratings might be more susceptible to immune evasion. We evaluated the score of immune cells and immune function by ssGSEA. The results revealed that the enrichment scores of multiple immune cells (B cells, DCs, iDCs, macrophages, mast cells, neutrophils, pDCs, T helper cells, TIL, and Treg cells) were elevated in the high-risk group (Figure 7A). Furthermore, immune function scores such as APC co-stimulation, CCR, check-point, MHC class 1, parainflammation, T cell co-stimulation, type I IFN response, and type II IFN response were also significantly elevated in the high-risk group (Figure 7B). These results demonstrate that ICD-related lncRNAs are involved in the regulation of immune cell function. Besides, immune cell proportion and function differed from risk groups, checkpoints and HLA genes were elevated with risk scores (Figures 7C,D). Above all, high-risk individuals exhibited immune-hot phenotypes, and low-risk individuals exhibited immune-cold characteristics. In this sense, immune checkpoint inhibitor (ICI) treatment may be more advantageous for high-risk individuals than low-risk ones.

### Comprehensive analyses of enriched pathways

**FIGURE 8:**
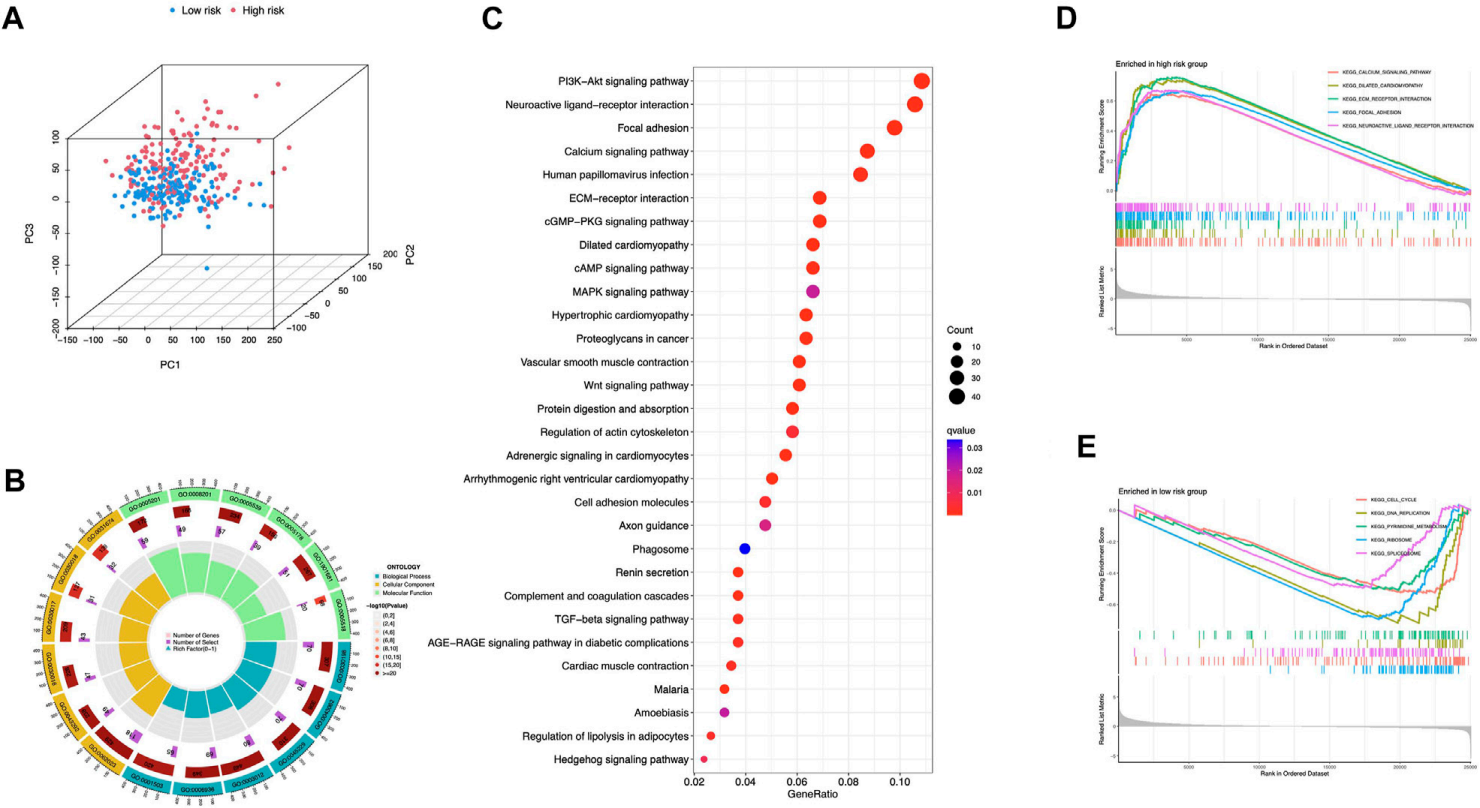
Comprehensive analyses of enriched pathways **(A)** Principal components analysis (PCA) analysis of risk groups based on the 5 ICD-associated lncRNAs. GO **(B)** and KEGG **(C)** enrichment of DEGs in groups with high and low risk. GSEA enrichment in the high-risk **(D)** and low-risk group **(E)**.

Principal Components Analysis (PCA) analysis suggested that STAD cases could be classified into two distinct clusters according to their risk scores (Figure 8A). 978 differentially expressed genes (DEGs) were then sorted out (adjusted *p*-value < 0.05, |log_2_FC| > 1) (Supplementary Figure S1). GO enrichment was shown in Figure 8B and Supplementary Table S1. DEGs were enriched in PI3K-Akt signaling pathway, MAPK signaling pathway, and Wnt signaling pathway (Figure 8C). The aberrant Wnt/β-catenin signaling pathway facilitates cancer stem cell renewal and differentiation, thus exerting crucial roles in tumorigenesis and therapy response (Zhang and Wang, 2020). Wnt pathway can also affect Treg cells, T-helper cells, dendritic cells, and other cytokine-expressing immunocytes. Activation of Wnt signaling results in increased resistance to immunotherapies (Li et al., 2019). In addition, pathways including calcium signaling, dilated cardiomyopathy, and ECM receptor interaction were selectively enriched in the high-risk group (Figure 8D). Low-risk group had cell cycle, DNA replication, and pyrimidine metabolism enrichments (Figure 8E).

### Somatic mutation

**FIGURE 9:**
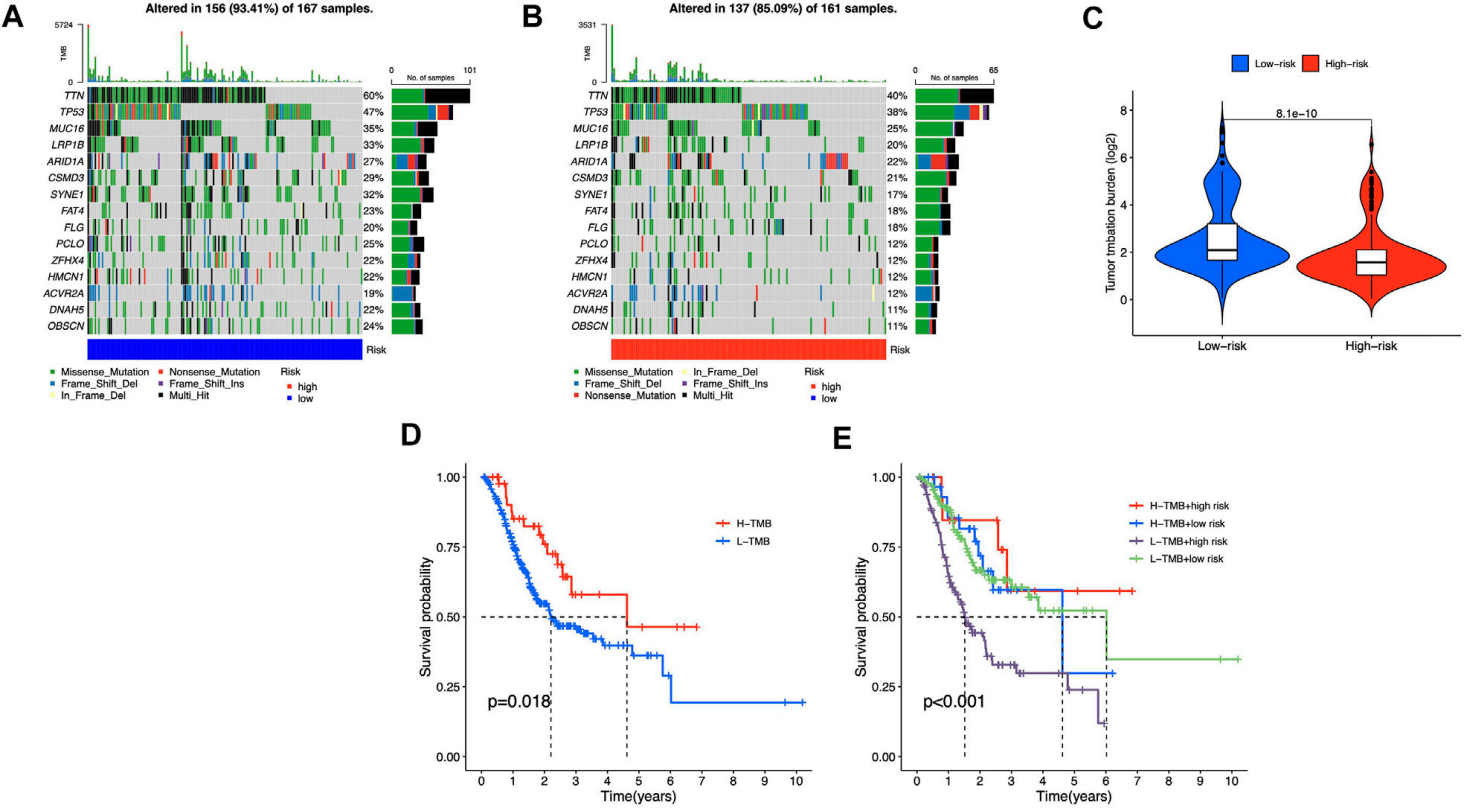
Somatic mutations in different risk groups **(A,B)** Oncoprint of genes with the most mutations **(C)** TMB of STAD cases in groups with high and low risk **(D,E)** The correlation between TMB and survival probability.

We identified differential mutations using Fisher’s exact test with a *p* value of 0.01 threshold, and diverse somatic mutation profiles were discovered in the two risk groups. TTN, TP53, MUC16, LRP1B, and ARID1A had most gene mutations, with TTN ranking first (Figures 9A,B). Low-risk group had greater tumor mutational burden (TMB) than high-risk group (Figure 9C). Besides, patients with higher TMB scores showed better OS in STAD cohorts (Figure 9D), and in the high-risk category, observations with low TMB scores had the poorest OS (Figure 9E). These findings showed that somatic mutations were connected with risk scores and survival probabilities; individuals in low-risk group with higher TMB scores may have a greater chance of surviving.

### Correlation analysis between the risk group and chemotherapeutics

**FIGURE 10:**
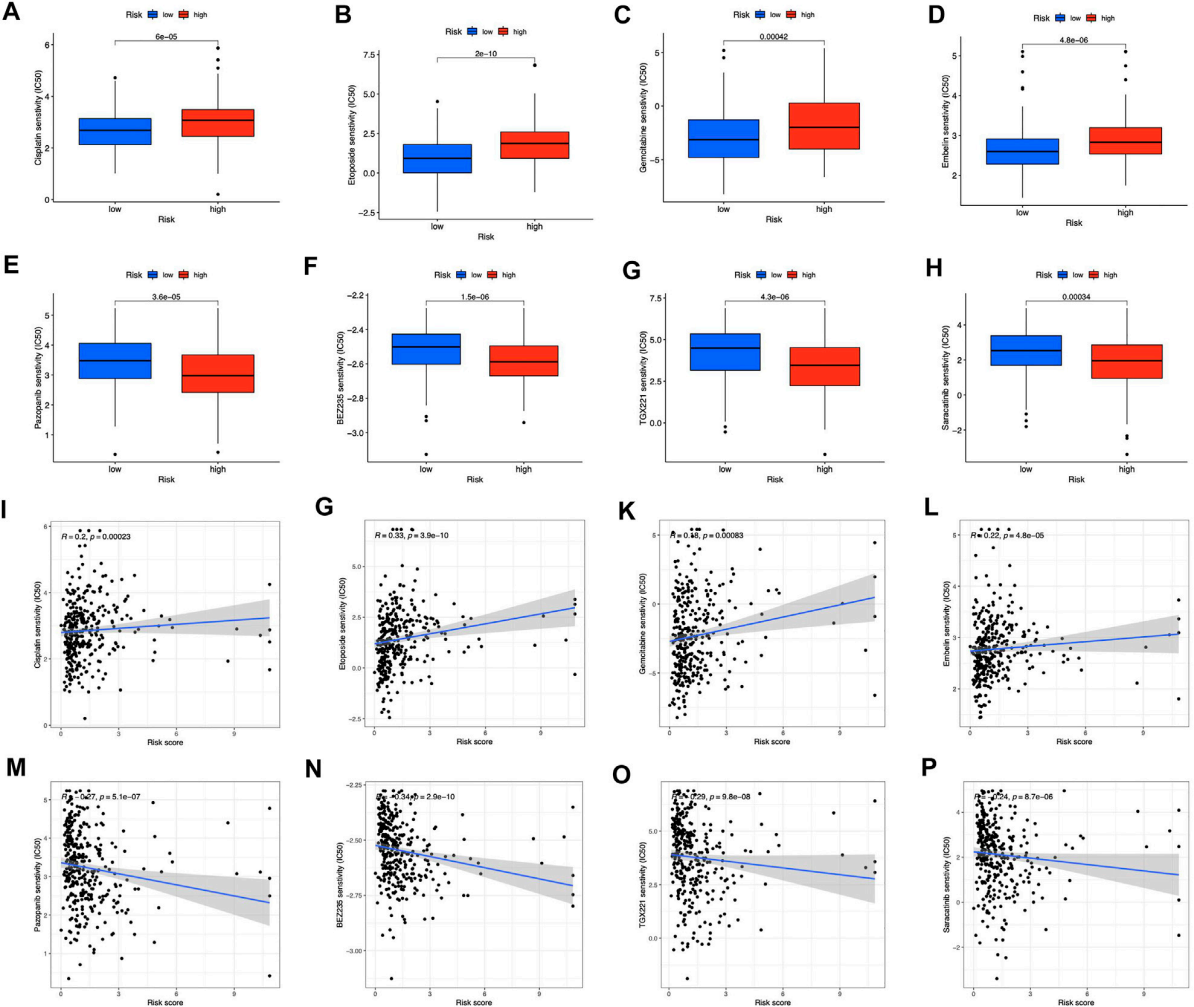
Prediction of drug sensitivity in risk groups **(A–D)** Drugs sensitive in low-risk groups **(E–H)** Drugs sensitive in high-risk groups **(I–P)** Correlation between sensitive drugs and risk scores.

For clinical application probability, we examined the drug sensitivity of the two risk groups. According to the results, gemcitabine, cisplatin, etoposide, and embelin were effective on the low-risk patients (Figures 10A–D). High-risk patients were more susceptible to pazopanib (Figure 10E), BEZ235 (Figure 10F), TGX221 (Figure 10G), and Saracatinib (Figure 10H). Besides, we found that all the sensitive drugs correlated with risk scores (Figures 10I–P). Gemcitabine, cisplatin, etoposide, and pazopanib are first-line treatment antitumor drugs. Currently, embelin is used for purely scientific purposes and is not available for general use. The three additional sensitive medications in the high-risk category have not undergone medical trials. They may be promising in the future.

## Discussion

The absence of potent antitumor initiators and precise tumor-targeting therapeutic agents presently hampers STAD precision treatment. Studies have indicated that immunogenic cell death (ICD) may be effectively regulated in order to enhance the therapeutic effects of STAD (Xu et al., 2013; Petersen et al., 2021; Xiao et al., 2022). For the treatment of STAD, it offers great promise to combine immunogenic therapeutics with novel immunotherapeutic regimens (Zeng et al., 2021). Immunotherapy, which includes immune checkpoint activity blocking, anti-cancer T cell response activation, and adoptive cellular therapy to prime the patient’s own lymphocytes to attack cancer cells, has become a potent clinical technique for treating cancer (Farkona et al., 2016). However, certain cancer treatments generate apoptotic cell death that is immunologically silent and can also damage the immune system, allowing cancer to recur (Guo et al., 2013). Eliciting ICD could potentially turn these dying cancer cells into “vaccines” that promote anticancer immunity by maturing DCs, activating CTLs, and increasing the cytotoxic activity of NK cells (Krysko et al., 2012; Kroemer et al., 2013; Ahmed and Tait, 2020). When effectively induced, ICD could stimulate the body’s cytotoxic lymphocytes to eliminate tumor cells and eventually achieve long-term anticancer immunity against cancer recurrence and spread. When combined with ICD induction, patients with weakly immunogenic malignancies may be made sensitive to checkpoint inhibitors (Showalter et al., 2017). As a consequence, biomarkers related to ICD could assist in the differentiation of STAD sufferers who might benefit from immunotherapy.

STAD pathology involves the regulation of various cellular processes by lncRNAs. However, lncRNAs associated with ICD may affect STAD pathology and survival. According to our study, we found that lncRNAs that are associated with ICD can affect the prognosis of STAD patients. We developed and verified a predictive risk signature utilizing five chosen lncRNAs associated with the ICD. The STAD cohorts were classified depending on risk scores. This risk signature predicted OS accurately. It was correlated to immune cell infiltration, immunological checkpoints, TMB and TIDE scores, as well as pharmacological responsiveness. The usage of this risk model may be advantageous for STAD sufferers.

The current investigation identified five prognosis-related lncRNAs (AC131391.1, PVT1, LINC00592, AL139147.1, and VCAN-AS1), all of which were upregulated in STAD samples (Figure 2A). Afterward, an independent prognosis model was established, which might be applied for STAD prognostic prediction. Our suggested model’s AUCs for 1-, 3-, and 5-year OS were 0.696, 0.640, and 0.649, demonstrating that the risk signature had outstanding predictive potential in STAD. Furthermore, the nomogram combining risk scores and clinical characteristics enhances the ability to predict the prognosis of STAD.

Increasing numbers of non-coding RNAs have been identified in gastric cancer that is implicated in drug resistance development (Wei et al., 2020). LncRNA PVT1 activates Wnt/-catenin and autophagy to enhance gemcitabine resistance in pancreatic cancer (Zhou et al., 2020). Zhao et al. demonstrated that PVT1 promotes gastric cancer angiogenesis through STAT3/VEGFA activation (Zhao et al., 2018); PCVT1’s impacts on cell proliferation, migration, invasion, and apoptosis raise cancer risk (Pan et al., 2018; Ghafouri-Fard et al., 2019; Wang et al., 2020), suggesting that it could be targeted in cancer therapy. LINC00592 was involved in a prognostic model of gastric cancer (Cheng et al., 2019), and Yuan et al. found that LINC00592 might activate the cervical cancer progression (Yuan et al., 2019). VCAN-AS1 interacts with eIF4A3 to downregulate TP53 expression and might be a potential cancer therapy target (Feng et al., 2020); AC131391.1 and AL139147.1 were firstly discovered. Whereas AL139147.1 showed a novel positive correlation with ENTPD1 (CD39) and IL1R1 (Figure 3F), which are involved in tumor immune cells infiltration and tumor microenvironment alteration (Krishna et al., 2020; Zhang et al., 2020); AC131391.1 correlated with LY96, which plays a vital role in tumorigenesis by modulating host immunity (Nie et al., 2022). The newly discovered lncRNA knowledge about ICD might help us bring a breakthrough into clinical practice by improving our mechanistic understanding of STAD.

According to the tumor immune editing hypothesis, immune responses can be evaded by targeting fewer immunogenic cancer cells in immunocompetent hosts (Dunn et al., 2002). In line with expectations, the two risk groups have dissimilar immunological microenvironments. High-risk patients showed greater immunological infiltrates and higher immune scores. Tregs are immunoregulatory cells that suppress cytokine growth and production. Inappropriate or dysfunctional Treg production may impair the immune system (Hou et al., 2014). In recent decades, tumor-infiltrating Treg cells have been linked to immune evasion (Kindlund et al., 2017). Moreover, Tregs are associated with poor outcomes in gastric cancer (Yuan et al., 2010). Besides, High-risk patients had more Tregs (Figure 7A), which may promote tumor development and immunity escape by secreting immunosuppressive cytokines, stimulating antiapoptotic molecules, and promoting tumor cell survival (Mao et al., 2017; Gao et al., 2019). Most immune cells correlate positively with risk ratings, and high-risk groups have higher TME scores. So, immunotherapy may be a good option for high-risk patients. We evaluated immunological checkpoint and HLA gene expression. The findings validated our hypothesis and gave some context for immune checkpoint inhibitor (ICI) therapy in STAD patients.

To assess the model’s effectiveness in immunotherapy, we used TIDE scores to predict immune escape. Our findings suggested that low-risk individuals, had lower TIDE scores, might benefit better from ICI therapy in our risk model. Aside from immunotherapy, we discovered an association between the patients’ signatures and chemotherapy. Low-risk patients were sensitive to first-line anticancer medicines (including etoposide, cisplatin, and gemcitabine) (Macdonald and Havlin, 1992; Qian et al., 2019; Sugisawa et al., 2020). Low-risk patients react better to chemotherapy and targeted medicines, which is crucial for tailored tumor treatments. Furthermore, the low-risk TCGA-STAD cohort exhibited pathway enrichment in the cell cycle. Sensitive drugs’ antitumor actions are also aimed mainly towards the cell cycle and DNA replication (Macdonald and Havlin, 1992; Qian et al., 2019). Embelin, which targets PI3-kinase/AKT (Prabhu et al., 2018), demonstrated sensitivity in low-risk group. Pazopanib, targeting VEGFR2 and PDGFR, showed sensitivity to the high-risk group (Kim et al., 2014). Among the newly developed inhibitors of PI3K and mTOR, BEZ235 has shown promising results against gastric cancer chemotherapy (Li et al., 2018). Saracatinib inhibits stomach cancer cell proliferation, migration, and invasion (HJ Nam et al., 2013). TGX221 is a new, highly selective inhibitor for clear cell renal cell cancer (Feng et al., 2015). These drugs may contribute to effective chemotherapy in the high-risk group. Our findings may offer prospective treatment alternatives for patients suffering from STAD and affect individualized tumor therapy.

This study’s major findings and implications can be summarized as follows. For starters, this is the first in-depth study of a 5 ICD-related lncRNA signature for predicting STAD patient prognosis. Second, according to our model, TIDE scores and immune infiltration were connected to the risk score, as well as TMB alteration, suggesting a tumor status and immune response, which could represent potential targets for therapeutic intervention. Third, predicting sensitive drugs may contribute to the improvement of STAD immunotherapy and provide personalized treatment options for individuals with STAD. Despite our multiple verification efforts, there are still some limitations. The model was only verified by TCGA for the lack of lncRNA data from other sources. A larger sample size is necessary for definitive conclusions, and the prediction model should be validated externally and practically before it is applied to clinical patients.

## Conclusion

Developing an exploitable treatment approach based on ICD-related lncRNAs and a unique risk model might assist STAD patients. This would improve individual therapy and patients’ prognoses. By targeting at lncRNAs associated with ICD, it may be possible to overcome systemic treatment failures and expand immunotherapy. These findings may have implications for immunotherapy and chemotherapy for STAD patients.

## Data Availability

The original contributions presented in the study are included in the article/Supplementary Material, further inquiries can be directed to the corresponding author.
