# Supplementary figures and images for: Prognostic value of antitumor drug targets prediction using integrated bioinformatic analysis for immunogenic cell death-related lncRNA model based on stomach adenocarcinoma characteristics and tumor immune microenvironment
Source: Front Pharmacol. 2022 Oct 14;13:1022294. doi: 10.3389/fphar.2022.1022294 (PMC9614277; doi:10.3389/fphar.2022.1022294)

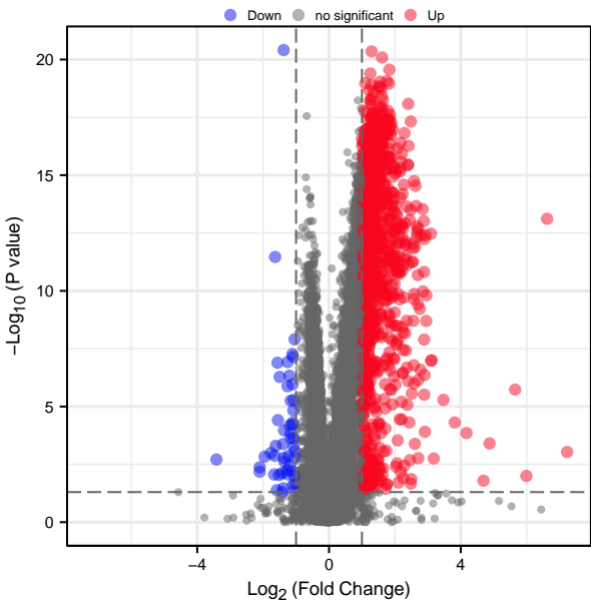

Supplement: Supplementary file 1 [file DataSheet2.PDF]
