# Supplementary material for: Prognostic value of antitumor drug targets prediction using integrated bioinformatic analysis for immunogenic cell death-related lncRNA model based on stomach adenocarcinoma characteristics and tumor immune microenvironment
Source: Front Pharmacol. 2022 Oct 14;13:1022294. doi: 10.3389/fphar.2022.1022294 (PMC9614277; doi:10.3389/fphar.2022.1022294)
Supplement: Supplementary file 2 [file DataSheet1.PDF]

Supplementary Table 1. GO enrichment of DEGs in high- and low-risk groups

ONTOLOGY	ID	Description	BgRatio	pvalue	qvalue	Count
BP	GO:0030198	extracellular ı	307/18800	3.93E-31	8.22E-28	70
BP	GO:0043062	extracellular :	308/18800	4.88E-31	8.22E-28	70
BP	GO:0045229	external enca	310/18800	7.52E-31	8.44E-28	70
BP	GO:0003012	muscle syster	449/18800	1.19E-27	1.00E-24	80
BP	GO:0006936	muscle contra	349/18800	1.01E-26	6.82E-24	69
BP	GO:0001503	ossification	420/18800	2.97E-19	1.67E-16	65
BP	GO:0031589	cell-substrate	364/18800	1.28E-16	6.16E-14	56
BP	GO:0061448	connective tis	260/18800	1.61E-15	6.80E-13	45
BP	GO:0042692	muscle cell di	387/18800	2.03E-15	7.58E-13	56
BP	GO:0060537	muscle tissue	405/18800	3.93E-15	1.32E-12	57
BP	GO:0016055	Wnt signaling	452/18800	1.08E-14	3.32E-12	60
BP	GO:0198738	cell-cell signa	454/18800	1.33E-14	3.72E-12	60
BP	GO:0051216	cartilage deve	195/18800	2.81E-14	7.01E-12	37
BP	GO:0006939	smooth musc	112/18800	2.91E-14	7.01E-12	28
BP	GO:0003018	vascular proc	263/18800	5.51E-14	1.24E-11	43
BP	GO:0030199	collagen fibril	62/18800	6.80E-14	1.34E-11	21
BP	GO:0007517	muscle organ	334/18800	7.09E-14	1.34E-11	49
BP	GO:0008016	regulation of	211/18800	7.15E-14	1.34E-11	38
BP	GO:0030111	regulation of	336/18800	8.95E-14	1.59E-11	49
BP	GO:1903522	regulation of	260/18800	1.66E-13	2.79E-11	42
BP	GO:0090257	regulation of	250/18800	1.93E-13	3.09E-11	41
BP	GO:0061564	axon develop	479/18800	4.91E-13	7.52E-11	59
BP	GO:0060047	heart contrac	247/18800	5.81E-13	8.51E-11	40
BP	GO:0030178	negative regu	174/18800	7.13E-13	1.00E-10	33
BP	GO:0060485	mesenchyme	301/18800	1.55E-12	2.09E-10	44
BP	GO:0003015	heart process	257/18800	2.15E-12	2.79E-10	40
BP	GO:0007160	cell-matrix ac	235/18800	2.33E-12	2.90E-10	38
BP	GO:0055001	muscle cell de	183/18800	3.07E-12	3.69E-10	33
BP	GO:0035637	multicellular	164/18800	3.90E-12	4.52E-10	31
BP	GO:0033002	muscle cell pı	233/18800	7.91E-12	8.88E-10	37
BP	GO:0060828	regulation of	261/18800	1.48E-11	1.61E-09	39
BP	GO:0010810	regulation of	217/18800	1.86E-11	1.95E-09	35
BP	GO:0060070	canonical Wn	313/18800	2.27E-11	2.31E-09	43
BP	GO:0051146	striated musc	281/18800	3.77E-11	3.74E-09	40
BP	GO:0006937	regulation of	170/18800	5.25E-11	5.05E-09	30
BP	GO:0030282	bone mineral	121/18800	6.38E-11	5.97E-09	25
BP	GO:0042060	wound healir	429/18800	7.27E-11	6.62E-09	51
BP	GO:0007409	axonogenesis	430/18800	7.92E-11	7.01E-09	51
BP	GO:0001649	osteoblast dil	241/18800	9.12E-11	7.74E-09	36
BP	GO:0090287	regulation of	314/18800	9.19E-11	7.74E-09	42
BP	GO:0019932	second-mess	303/18800	1.07E-10	8.82E-09	41
BP	GO:0007178	transmembra	368/18800	1.19E-10	9.53E-09	46

BP	GO:0061337	cardiac condu	98/18800	1.64E-10	1.28E-08	22
BP	GO:0055074	calcium ion h	468/18800	1.87E-10	1.43E-08	53
BP	GO:0048660	regulation of	170/18800	2.55E-10	1.91E-08	29
BP	GO:0001655	urogenital sy:	352/18800	3.03E-10	2.22E-08	44
BP	GO:0048659	smooth musc	173/18800	3.92E-10	2.81E-08	29
BP	GO:0090090	negative regu	142/18800	4.40E-10	3.09E-08	26
BP	GO:0042391	regulation of	425/18800	5.01E-10	3.44E-08	49
BP	GO:0006874	cellular calciu	456/18800	6.46E-10	4.35E-08	51
BP	GO:0042698	ovulation cyc	70/18800	7.14E-10	4.71E-08	18
BP	GO:0045667	regulation of	136/18800	8.73E-10	5.65E-08	25
BP	GO:0032963	collagen meta	101/18800	1.91E-09	1.17E-07	21
BP	GO:0035296	regulation of	141/18800	1.91E-09	1.17E-07	25
BP	GO:0097746	blood vessel (	141/18800	1.91E-09	1.17E-07	25
BP	GO:0035150	regulation of	142/18800	2.23E-09	1.34E-07	25
BP	GO:0034329	cell junction a	420/18800	3.02E-09	1.75E-07	47
BP	GO:0014706	striated musc	248/18800	3.02E-09	1.75E-07	34
BP	GO:0060348	bone develop	225/18800	3.52E-09	2.01E-07	32
BP	GO:0051480	regulation of	356/18800	4.46E-09	2.51E-07	42
BP	GO:0048705	skeletal syste	228/18800	4.91E-09	2.71E-07	32
BP	GO:0090092	regulation of	267/18800	5.85E-09	3.18E-07	35
BP	GO:0008217	regulation of	183/18800	6.52E-09	3.49E-07	28
BP	GO:0048762	mesenchyma	244/18800	7.27E-09	3.82E-07	33
BP	GO:0048565	digestive trac	130/18800	8.83E-09	4.54E-07	23
BP	GO:0048771	tissue remod	174/18800	8.90E-09	4.54E-07	27
BP	GO:0048738	cardiac muscl	234/18800	9.35E-09	4.70E-07	32
BP	GO:0072503	cellular divale	494/18800	9.99E-09	4.95E-07	51
BP	GO:0072001	renal system	312/18800	1.02E-08	5.00E-07	38
BP	GO:0055123	digestive syst	142/18800	1.08E-08	5.17E-07	24
BP	GO:0006940	regulation of	65/18800	1.25E-08	5.93E-07	16
BP	GO:0010927	cellular comp	112/18800	1.34E-08	6.28E-07	21
BP	GO:0006941	striated musc	178/18800	1.47E-08	6.76E-07	27
BP	GO:0019934	cGMP-mediat	36/18800	2.03E-08	9.22E-07	12
BP	GO:0002027	regulation of	105/18800	2.25E-08	1.01E-06	20
BP	GO:0031214	biomineral tis	170/18800	2.28E-08	1.01E-06	26
BP	GO:0033627	cell adhesion	86/18800	2.44E-08	1.07E-06	18
BP	GO:0110148	biomineraliza	172/18800	2.91E-08	1.26E-06	26
BP	GO:0007204	positive regul	325/18800	3.11E-08	1.32E-06	38
BP	GO:0007411	axon guidanc	234/18800	3.37E-08	1.42E-06	31
BP	GO:0030278	regulation of	118/18800	3.50E-08	1.45E-06	21
BP	GO:0097485	neuron proje	235/18800	3.73E-08	1.53E-06	31
BP	GO:0061035	regulation of	70/18800	3.90E-08	1.58E-06	16
BP	GO:0031032	actomyosin s	199/18800	4.23E-08	1.69E-06	28
BP	GO:0050804	modulation o	429/18800	4.68E-08	1.85E-06	45
BP	GO:0001822	kidney develo	303/18800	4.75E-08	1.86E-06	36

BP	GO:0099177	regulation of	430/18800	5.01E-08	1.94E-06	45
BP	GO:0071560	cellular respo	252/18800	5.61E-08	2.15E-06	32
BP	GO:0010811	positive regul	122/18800	6.39E-08	2.39E-06	21
BP	GO:0050808	synapse orga	419/18800	6.39E-08	2.39E-06	44
BP	GO:0001667	ameboidal-ty	480/18800	7.34E-08	2.69E-06	48
BP	GO:0048662	negative regu	73/18800	7.34E-08	2.69E-06	16
BP	GO:0048251	elastic fiber a	11/18800	8.15E-08	2.92E-06	7
BP	GO:1901342	regulation of	351/18800	8.16E-08	2.92E-06	39
BP	GO:0019935	cyclic-nucleot	83/18800	8.34E-08	2.96E-06	17
BP	GO:1904062	regulation of	352/18800	8.81E-08	3.09E-06	39
BP	GO:0071559	response to t	258/18800	9.76E-08	3.39E-06	32
BP	GO:0022602	ovulation cyc	49/18800	1.12E-07	3.83E-06	13
BP	GO:0030239	myofibril asse	66/18800	1.12E-07	3.83E-06	15
BP	GO:0050673	epithelial cell	443/18800	1.20E-07	4.04E-06	45
BP	GO:0060048	cardiac muscl	138/18800	1.29E-07	4.26E-06	22
BP	GO:0061138	morphogenes	185/18800	1.31E-07	4.26E-06	26
BP	GO:0071772	response to B	173/18800	1.32E-07	4.26E-06	25
BP	GO:0071773	cellular respo	173/18800	1.32E-07	4.26E-06	25
BP	GO:0086065	cell communi	58/18800	1.33E-07	4.26E-06	14
BP	GO:0060840	artery develo	106/18800	1.39E-07	4.38E-06	19
BP	GO:0055002	striated musc	67/18800	1.39E-07	4.38E-06	15
BP	GO:0045765	regulation of	345/18800	1.50E-07	4.68E-06	38
BP	GO:0001763	morphogenes	199/18800	1.57E-07	4.86E-06	27
BP	GO:0042063	gliogenesis	291/18800	1.68E-07	5.14E-06	34
BP	GO:2000027	regulation of	129/18800	1.72E-07	5.21E-06	21
BP	GO:0034764	positive regul	213/18800	1.82E-07	5.48E-06	28
BP	GO:0035051	cardiocyte dif	153/18800	2.01E-07	6.00E-06	23
BP	GO:0032970	regulation of	393/18800	2.15E-07	6.35E-06	41
BP	GO:0003007	heart morpho	254/18800	2.22E-07	6.51E-06	31
BP	GO:0007369	gastrulation	190/18800	2.24E-07	6.51E-06	26
BP	GO:1903034	regulation of	166/18800	2.32E-07	6.67E-06	24
BP	GO:0001508	action potent	143/18800	2.44E-07	6.98E-06	22
BP	GO:0040013	negative regu	396/18800	2.64E-07	7.46E-06	41
BP	GO:0061041	regulation of	133/18800	2.92E-07	8.20E-06	21
BP	GO:0007188	adenylate cyc	244/18800	2.96E-07	8.24E-06	30
BP	GO:0051271	negative regu	369/18800	3.04E-07	8.39E-06	39
BP	GO:0007596	blood coagula	221/18800	3.95E-07	1.08E-05	28
BP	GO:0007044	cell-substrate	92/18800	3.99E-07	1.08E-05	17
BP	GO:0010959	regulation of	403/18800	4.20E-07	1.13E-05	41
BP	GO:0085029	extracellular	46/18800	4.22E-07	1.13E-05	12
BP	GO:0030336	negative regu	346/18800	4.57E-07	1.21E-05	37
BP	GO:2000146	negative regu	361/18800	4.77E-07	1.25E-05	38
BP	GO:0090288	negative regu	115/18800	5.20E-07	1.36E-05	19
BP	GO:0030509	BMP signaling	162/18800	5.73E-07	1.48E-05	23

BP	GO:0098742	cell-cell adhe	279/18800	5.85E-07	1.50E-05	32
BP	GO:0035265	organ growth	175/18800	6.24E-07	1.58E-05	24
BP	GO:0050817	coagulation	226/18800	6.26E-07	1.58E-05	28
BP	GO:0007599	hemostasis	227/18800	6.85E-07	1.72E-05	28
BP	GO:0090066	regulation of	487/18800	7.07E-07	1.76E-05	46
BP	GO:0007229	integrin-medi	107/18800	7.85E-07	1.94E-05	18
BP	GO:0030850	prostate glan	49/18800	8.87E-07	2.16E-05	12
BP	GO:0048546	digestive trac	49/18800	8.87E-07	2.16E-05	12
BP	GO:0010975	regulation of	431/18800	9.59E-07	2.32E-05	42
BP	GO:0150115	cell-substrate	98/18800	1.01E-06	2.44E-05	17
BP	GO:0090263	positive regul	109/18800	1.04E-06	2.48E-05	18
BP	GO:0048608	reproductive	433/18800	1.08E-06	2.57E-05	42
BP	GO:0030177	positive regul	144/18800	1.13E-06	2.66E-05	21
BP	GO:0002062	chondrocyte	110/18800	1.19E-06	2.79E-05	18
BP	GO:0061458	reproductive	436/18800	1.30E-06	3.01E-05	42
BP	GO:0048844	artery morph	79/18800	1.33E-06	3.08E-05	15
BP	GO:0010763	positive regul	15/18800	1.36E-06	3.12E-05	7
BP	GO:0032330	regulation of	51/18800	1.41E-06	3.20E-05	12
BP	GO:0016049	cell growth	484/18800	1.44E-06	3.25E-05	45
BP	GO:0060284	regulation of	500/18800	1.47E-06	3.30E-05	46
BP	GO:0060191	regulation of	90/18800	1.50E-06	3.30E-05	16
BP	GO:0001704	formation of	123/18800	1.50E-06	3.30E-05	19
BP	GO:0022612	gland morpho	123/18800	1.50E-06	3.30E-05	19
BP	GO:0090101	negative regu	135/18800	1.57E-06	3.44E-05	20
BP	GO:0007611	learning or m	264/18800	1.59E-06	3.46E-05	30
BP	GO:0014909	smooth musc	91/18800	1.74E-06	3.77E-05	16
BP	GO:0086009	membrane re	52/18800	1.76E-06	3.77E-05	12
BP	GO:1904064	positive regul	148/18800	1.78E-06	3.79E-05	21
BP	GO:0070252	actin-mediate	103/18800	2.08E-06	4.40E-05	17
BP	GO:0003231	cardiac ventri	126/18800	2.17E-06	4.56E-05	19
BP	GO:0072132	mesenchyme	53/18800	2.18E-06	4.56E-05	12
BP	GO:0034765	regulation of	476/18800	2.19E-06	4.56E-05	44
BP	GO:0031644	regulation of	138/18800	2.23E-06	4.61E-05	20
BP	GO:1901888	regulation of	201/18800	2.30E-06	4.70E-05	25
BP	GO:0010881	regulation of	22/18800	2.30E-06	4.70E-05	8
BP	GO:0007200	phospholipas	104/18800	2.39E-06	4.81E-05	17
BP	GO:0014812	muscle cell m	104/18800	2.39E-06	4.81E-05	17
BP	GO:0019722	calcium-medi	202/18800	2.52E-06	5.05E-05	25
BP	GO:0014910	regulation of	83/18800	2.56E-06	5.07E-05	15
BP	GO:0048041	focal adhesio	83/18800	2.56E-06	5.07E-05	15
BP	GO:0010092	specification	37/18800	2.75E-06	5.42E-05	10
BP	GO:0048754	branching mo	153/18800	3.06E-06	5.97E-05	21
BP	GO:0048545	response to s	330/18800	3.07E-06	5.97E-05	34
BP	GO:0045669	positive regul	74/18800	3.13E-06	6.06E-05	14

BP	GO:0043270	positive regul	273/18800	3.18E-06	6.12E-05	30
BP	GO:0061037	negative regu	30/18800	3.35E-06	6.41E-05	9
BP	GO:1902903	regulation of	376/18800	3.37E-06	6.41E-05	37
BP	GO:0003205	cardiac cham	167/18800	3.58E-06	6.77E-05	22
BP	GO:2001257	regulation of	180/18800	3.66E-06	6.87E-05	23
BP	GO:0048645	animal organ	65/18800	3.67E-06	6.87E-05	13
BP	GO:0086003	cardiac muscl	75/18800	3.70E-06	6.88E-05	14
BP	GO:0048557	embryonic di	17/18800	3.82E-06	7.06E-05	7
BP	GO:0010466	negative regu	262/18800	4.01E-06	7.37E-05	29
BP	GO:1903169	regulation of	156/18800	4.19E-06	7.67E-05	21
BP	GO:0032412	regulation of	263/18800	4.32E-06	7.86E-05	29
BP	GO:0010517	regulation of	66/18800	4.39E-06	7.95E-05	13
BP	GO:0010880	regulation of	31/18800	4.54E-06	8.18E-05	9
BP	GO:0060349	bone morpho	98/18800	4.78E-06	8.56E-05	16
BP	GO:0072006	nephron deve	145/18800	4.85E-06	8.64E-05	20
BP	GO:0086001	cardiac muscl	77/18800	5.11E-06	9.05E-05	14
BP	GO:0003002	regionalizatio	354/18800	5.56E-06	9.80E-05	35
BP	GO:0001558	regulation of	415/18800	5.59E-06	9.80E-05	39
BP	GO:0007389	pattern speci	463/18800	5.96E-06	0.00010401	42
BP	GO:0060193	positive regul	68/18800	6.22E-06	0.00010789	13
BP	GO:0007179	transforming	200/18800	6.82E-06	0.00011778	24
BP	GO:0014068	positive regul	79/18800	6.98E-06	0.00011993	14
BP	GO:0070588	calcium ion tr	314/18800	7.47E-06	0.00012767	32
BP	GO:0001837	epithelial to r	162/18800	7.65E-06	0.00013002	21
BP	GO:0048333	mesodermal	33/18800	8.04E-06	0.00013602	9
BP	GO:1901019	regulation of	91/18800	8.34E-06	0.00014046	15
BP	GO:0034767	positive regul	163/18800	8.42E-06	0.00014112	21
BP	GO:0003206	cardiac cham	126/18800	8.71E-06	0.00014528	18
BP	GO:0022898	regulation of	273/18800	8.97E-06	0.00014872	29
BP	GO:0030500	regulation of	81/18800	9.44E-06	0.00015502	14
BP	GO:0055013	cardiac muscl	81/18800	9.44E-06	0.00015502	14
BP	GO:0007157	heterophilic c	51/18800	9.55E-06	0.00015541	11
BP	GO:0045778	positive regul	51/18800	9.55E-06	0.00015541	11
BP	GO:0010882	regulation of	26/18800	9.64E-06	0.00015607	8
BP	GO:0048706	embryonic sk	127/18800	9.75E-06	0.00015707	18
BP	GO:2001259	positive regul	71/18800	1.02E-05	0.00016368	13
BP	GO:0086019	cell-cell signa	34/18800	1.05E-05	0.00016767	9
BP	GO:0072073	kidney epithe	140/18800	1.06E-05	0.00016767	19
BP	GO:0090130	tissue migrati	366/18800	1.15E-05	0.00018214	35
BP	GO:0050890	cognition	306/18800	1.16E-05	0.00018309	31
BP	GO:0030510	regulation of	105/18800	1.19E-05	0.0001857	16
BP	GO:0003208	cardiac ventri	72/18800	1.20E-05	0.0001857	13
BP	GO:1903036	positive regul	72/18800	1.20E-05	0.0001857	13
BP	GO:0032411	positive regul	117/18800	1.22E-05	0.00018914	17

BP	GO:0032956	regulation of	352/18800	1.24E-05	0.00019072	34
BP	GO:0050900	leukocyte mi<br>g	384/18800	1.34E-05	0.00020474	36
BP	GO:0032414	positive regul	106/18800	1.34E-05	0.00020474	16
BP	GO:0010762	regulation of	35/18800	1.36E-05	0.00020549	9
BP	GO:0014808	release of sec	35/18800	1.36E-05	0.00020549	9
BP	GO:0048562	embryonic or	294/18800	1.39E-05	0.00020955	30
BP	GO:0010951	negative regu	251/18800	1.44E-05	0.0002159	27
BP	GO:0051893	regulation of	63/18800	1.46E-05	0.0002173	12
BP	GO:0090109	regulation of	63/18800	1.46E-05	0.0002173	12
BP	GO:0007548	sex differenti	281/18800	1.56E-05	0.00023014	29
BP	GO:0086002	cardiac muscl	54/18800	1.70E-05	0.00025064	11
BP	GO:0086010	membrane de	36/18800	1.74E-05	0.00025435	9
BP	GO:1903514	release of sec	36/18800	1.74E-05	0.00025435	9
BP	GO:0007612	learning	145/18800	1.76E-05	0.00025485	19
BP	GO:0010171	body morphc	45/18800	1.84E-05	0.00026525	10
BP	GO:0008406	gonad develo	226/18800	1.84E-05	0.00026525	25
BP	GO:1904705	regulation of	86/18800	1.92E-05	0.00027438	14
BP	GO:0055007	cardiac muscl	121/18800	1.92E-05	0.00027438	17
BP	GO:0001706	endoderm fo	55/18800	2.05E-05	0.00028972	11
BP	GO:0010518	positive regul	55/18800	2.05E-05	0.00028972	11
BP	GO:0006816	calcium ion tr	424/18800	2.15E-05	0.00030311	38
BP	GO:0052547	regulation of	456/18800	2.16E-05	0.00030311	40
BP	GO:0061045	negative regu	76/18800	2.20E-05	0.00030467	13
BP	GO:0055006	cardiac cell de	87/18800	2.20E-05	0.00030467	14
BP	GO:1990874	vascular asso	87/18800	2.20E-05	0.00030467	14
BP	GO:0001823	mesonephros	99/18800	2.37E-05	0.00032725	15
BP	GO:0010812	negative regu	66/18800	2.40E-05	0.00032818	12
BP	GO:0032835	glomerulus de	66/18800	2.40E-05	0.00032818	12
BP	GO:0043410	positive regul	491/18800	2.46E-05	0.00033526	42
BP	GO:0046849	bone remode	88/18800	2.51E-05	0.00034101	14
BP	GO:0046660	female sex di	124/18800	2.66E-05	0.00035973	17
BP	GO:0045137	development	231/18800	2.67E-05	0.00036029	25
BP	GO:0050919	negative cher	47/18800	2.75E-05	0.00036942	10
BP	GO:0086012	membrane de	22/18800	2.76E-05	0.00036942	7
BP	GO:0032409	regulation of	305/18800	2.82E-05	0.00037462	30
BP	GO:0007568	aging	163/18800	2.83E-05	0.00037462	20
BP	GO:0050678	regulation of	382/18800	2.86E-05	0.00037563	35
BP	GO:0050878	regulation of	382/18800	2.86E-05	0.00037563	35
BP	GO:0045785	positive regul	446/18800	2.93E-05	0.00038374	39
BP	GO:0070167	regulation of	101/18800	3.02E-05	0.00039445	15
BP	GO:0060325	face morphog	30/18800	3.09E-05	0.00040224	8
BP	GO:0150116	regulation of	68/18800	3.27E-05	0.0004241	12
BP	GO:0010761	fibroblast mig	48/18800	3.35E-05	0.00043163	10
BP	GO:0048568	embryonic or	449/18800	3.40E-05	0.00043572	39

BP	GO:0010717	regulation of	102/18800	3.40E-05	0.00043572	15
BP	GO:0030514	negative regu	58/18800	3.46E-05	0.00043961	11
BP	GO:0090303	positive regul	58/18800	3.46E-05	0.00043961	11
BP	GO:0060306	regulation of	39/18800	3.49E-05	0.00044056	9
BP	GO:0086091	regulation of	39/18800	3.49E-05	0.00044056	9
BP	GO:0051346	negative regu	371/18800	3.68E-05	0.00046273	34
BP	GO:0051147	regulation of	153/18800	3.76E-05	0.00047069	19
BP	GO:0034394	protein locali	69/18800	3.81E-05	0.00047258	12
BP	GO:0010522	regulation of	103/18800	3.83E-05	0.00047258	15
BP	GO:0110149	regulation of	103/18800	3.83E-05	0.00047258	15
BP	GO:0003151	outflow tract	80/18800	3.86E-05	0.00047258	13
BP	GO:0048644	muscle organ	80/18800	3.86E-05	0.00047258	13
BP	GO:0051279	regulation of	80/18800	3.86E-05	0.00047258	13
BP	GO:0031346	positive regul	341/18800	3.92E-05	0.00047786	32
BP	GO:0051924	regulation of	251/18800	3.97E-05	0.00048248	26
BP	GO:0060538	skeletal musc	167/18800	4.02E-05	0.00048663	20
BP	GO:0008038	neuron recog	49/18800	4.04E-05	0.00048773	10
BP	GO:0045165	cell fate comi	266/18800	4.06E-05	0.00048785	27
BP	GO:0045444	fat cell differe	237/18800	4.11E-05	0.00049173	25
BP	GO:0097553	calcium ion tr	154/18800	4.12E-05	0.00049173	19
BP	GO:0048880	sensory syste	389/18800	4.16E-05	0.00049474	35
BP	GO:1903035	negative regu	92/18800	4.19E-05	0.00049664	14
BP	GO:1903524	positive regul	40/18800	4.33E-05	0.00051217	9
BP	GO:0048511	rhythmic pro	297/18800	4.36E-05	0.00051349	29
BP	GO:0060562	epithelial tub	328/18800	4.50E-05	0.00052684	31
BP	GO:0007519	skeletal musc	155/18800	4.51E-05	0.00052684	19
BP	GO:0008585	female gonac	105/18800	4.81E-05	0.00055914	15
BP	GO:0030900	forebrain dev	376/18800	4.82E-05	0.00055914	34
BP	GO:0051960	regulation of	440/18800	4.83E-05	0.00055914	38
BP	GO:0050679	positive regul	211/18800	4.96E-05	0.00057162	23
BP	GO:0010644	cell communi	32/18800	5.15E-05	0.00059072	8
BP	GO:0060326	cell chemota	315/18800	5.16E-05	0.00059072	30
BP	GO:0001759	organ inducti	24/18800	5.20E-05	0.0005914	7
BP	GO:0043567	regulation of	24/18800	5.20E-05	0.0005914	7
BP	GO:0060402	calcium ion tr	171/18800	5.64E-05	0.00063931	20
BP	GO:0014831	gastro-intesti	11/18800	5.72E-05	0.00064594	5
BP	GO:0008347	glial cell migr	51/18800	5.81E-05	0.00065191	10
BP	GO:0045668	negative regu	51/18800	5.81E-05	0.00065191	10
BP	GO:0035567	non-canonica	72/18800	5.89E-05	0.00065902	12
BP	GO:0003073	regulation of	95/18800	6.02E-05	0.00067163	14
BP	GO:0097529	myeloid leuko	229/18800	6.45E-05	0.00071481	24
BP	GO:0045861	negative regu	350/18800	6.47E-05	0.00071481	32
BP	GO:0051899	membrane de	84/18800	6.53E-05	0.00071481	13
BP	GO:0048566	embryonic di	33/18800	6.54E-05	0.00071481	8

BP	GO:0030501	positive regul	42/18800	6.54E-05	0.00071481	9
BP	GO:0070296	sarcoplasmic	42/18800	6.54E-05	0.00071481	9
BP	GO:0030048	actin filamen	133/18800	6.59E-05	0.00071822	17
BP	GO:0051153	regulation of	96/18800	6.77E-05	0.00072883	14
BP	GO:0045123	cellular extra	73/18800	6.77E-05	0.00072883	12
BP	GO:0051145	smooth musc	73/18800	6.77E-05	0.00072883	12
BP	GO:0060415	muscle tissue	73/18800	6.77E-05	0.00072883	12
BP	GO:0048732	gland develo	431/18800	6.86E-05	0.00073565	37
BP	GO:0086064	cell communi	25/18800	6.95E-05	0.00074162	7
BP	GO:0150063	visual system	383/18800	6.96E-05	0.00074162	34
BP	GO:0031099	regeneration	188/18800	7.30E-05	0.00077568	21
BP	GO:0046545	development	109/18800	7.47E-05	0.00079104	15
BP	GO:0010712	regulation of	43/18800	7.95E-05	0.00083686	9
BP	GO:0071604	transforming	43/18800	7.95E-05	0.00083686	9
BP	GO:0010001	glial cell diffe	218/18800	8.22E-05	0.00085736	23
BP	GO:0060323	head morpho	34/18800	8.22E-05	0.00085736	8
BP	GO:0110110	positive regul	34/18800	8.22E-05	0.00085736	8
BP	GO:0060401	cytosolic calc	190/18800	8.51E-05	0.00088458	21
BP	GO:0001952	regulation of	123/18800	8.67E-05	0.00089812	16
BP	GO:0044703	multi-organis	205/18800	9.05E-05	0.000935	22
BP	GO:0060740	prostate glan	26/18800	9.16E-05	0.00093805	7
BP	GO:0048736	appendage de	177/18800	9.16E-05	0.00093805	20
BP	GO:0060173	limb develop	177/18800	9.16E-05	0.00093805	20
BP	GO:0072009	nephron epit	111/18800	9.23E-05	0.00094154	15
BP	GO:0001553	luteinization	12/18800	9.45E-05	0.0009606	5
BP	GO:0055017	cardiac muscl	87/18800	9.47E-05	0.0009606	13
BP	GO:0070372	regulation of	311/18800	9.95E-05	0.00100569	29
BP	GO:0021700	development	296/18800	0.00010195	0.00102781	28
BP	GO:0090075	relaxation of	35/18800	0.00010257	0.00103105	8
BP	GO:0003170	heart valve de	65/18800	0.00010308	0.00103305	11
BP	GO:0030324	lung develop	179/18800	0.00010714	0.00107054	20
BP	GO:0055093	response to h	19/18800	0.00011101	0.001095	6
BP	GO:0086014	atrial cardiac	19/18800	0.00011101	0.001095	6
BP	GO:0086026	atrial cardiac	19/18800	0.00011101	0.001095	6
BP	GO:0086066	atrial cardiac	19/18800	0.00011101	0.001095	6
BP	GO:1901343	negative regu	152/18800	0.00011121	0.001095	18
BP	GO:0003179	heart valve m	55/18800	0.00011361	0.00111533	10
BP	GO:0051384	response to g	139/18800	0.00011482	0.00112392	17
BP	GO:0055117	regulation of	77/18800	0.00011549	0.00112723	12
BP	GO:0072012	glomerulus va	27/18800	0.00011899	0.00115807	7
BP	GO:0001654	eye developm	379/18800	0.00012786	0.00124079	33
BP	GO:0007492	endoderm de	78/18800	0.00013117	0.00126922	12
BP	GO:0050920	regulation of	225/18800	0.00013263	0.00127973	23
BP	GO:0110053	regulation of	270/18800	0.00013358	0.00128521	26

BP	GO:0035987	endodermal (	46/18800	0.00013824	0.0013229	9
BP	GO:0048863	stem cell diff	211/18800	0.00013829	0.0013229	22
BP	GO:0007189	adenylate cyc	155/18800	0.00014297	0.00136378	18
BP	GO:0030323	respiratory tu	183/18800	0.00014521	0.00137348	20
BP	GO:0045766	positive regul	183/18800	0.00014521	0.00137348	20
BP	GO:1904018	positive regul	183/18800	0.00014521	0.00137348	20
BP	GO:0070509	calcium ion ir	91/18800	0.00015125	0.00142664	13
BP	GO:0033688	regulation of	28/18800	0.00015274	0.00142907	7
BP	GO:0060512	prostate glan	28/18800	0.00015274	0.00142907	7
BP	GO:0042474	middle ear m	20/18800	0.00015278	0.00142907	6
BP	GO:0060249	anatomical st	319/18800	0.00015471	0.00144246	29
BP	GO:0001658	branching inv	57/18800	0.00015507	0.00144246	10
BP	GO:0048009	insulin-like gr	37/18800	0.00015578	0.00144512	8
BP	GO:1900046	regulation of	68/18800	0.00015681	0.00145066	11
BP	GO:0044706	multi-multice	213/18800	0.00015857	0.00146287	22
BP	GO:0003197	endocardial c	47/18800	0.00016445	0.00150891	9
BP	GO:0032964	collagen bios	47/18800	0.00016445	0.00150891	9
BP	GO:0030168	platelet activ	130/18800	0.00016775	0.00153498	16
BP	GO:0031960	response to c	157/18800	0.00016832	0.00153599	18
BP	GO:0051017	actin filamen	158/18800	0.00018241	0.00166008	18
BP	GO:0043500	muscle adapt	118/18800	0.00018525	0.00168138	15
BP	GO:0003156	regulation of	29/18800	0.00019384	0.0017389	7
BP	GO:0061437	renal system	29/18800	0.00019384	0.0017389	7
BP	GO:0061440	kidney vascul	29/18800	0.00019384	0.0017389	7
BP	GO:0003044	regulation of	48/18800	0.00019468	0.0017389	9
BP	GO:0042311	vasodilation	48/18800	0.00019468	0.0017389	9
BP	GO:0060324	face developr	48/18800	0.00019468	0.0017389	9
BP	GO:0031102	neuron proje	59/18800	0.00020859	0.00185823	10
BP	GO:0001657	ureteric bud (	94/18800	0.00021081	0.00186811	13
BP	GO:0048704	embryonic sk	94/18800	0.00021081	0.00186811	13
BP	GO:0042310	vasoconstrict	82/18800	0.00021346	0.00188659	12
BP	GO:0051917	regulation of	14/18800	0.00022206	0.00195235	5
BP	GO:1901841	regulation of	14/18800	0.00022206	0.00195235	5
BP	GO:0010631	epithelial cell	358/18800	0.00022322	0.00195741	31
BP	GO:1900047	negative regu	49/18800	0.00022939	0.00198977	9
BP	GO:0009187	cyclic nucleot	39/18800	0.00022986	0.00198977	8
BP	GO:0014912	negative regu	39/18800	0.00022986	0.00198977	8
BP	GO:0045823	positive regul	39/18800	0.00022986	0.00198977	8
BP	GO:0071634	regulation of	39/18800	0.00022986	0.00198977	8
BP	GO:0048661	positive regul	95/18800	0.00023468	0.00201599	13
BP	GO:0072163	mesonephric	95/18800	0.00023468	0.00201599	13
BP	GO:0072164	mesonephric	95/18800	0.00023468	0.00201599	13
BP	GO:0014074	response to p	134/18800	0.00023888	0.0020445	16
BP	GO:0015844	monoamine t	83/18800	0.00023983	0.0020445	12

BP	GO:0048678	response to a	83/18800	0.00023983	0.0020445	12
BP	GO:1901654	response to k	190/18800	0.00024106	0.00204982	20
BP	GO:1990776	response to a	30/18800	0.00024341	0.00206464	7
BP	GO:0051209	release of sec	121/18800	0.00024511	0.00207308	15
BP	GO:0033674	positive regul	476/18800	0.00024564	0.00207308	38
BP	GO:0061572	actin filamen	162/18800	0.00024962	0.00210137	18
BP	GO:0010720	positive regul	297/18800	0.00025758	0.00216304	27
BP	GO:0090132	epithelium m	361/18800	0.00025843	0.00216475	31
BP	GO:0006942	regulation of	96/18800	0.00026083	0.00217363	13
BP	GO:0060419	heart growth	96/18800	0.00026083	0.00217363	13
BP	GO:0048638	regulation of	329/18800	0.00026143	0.00217363	29
BP	GO:0016525	negative regu	149/18800	0.000268	0.00221003	17
BP	GO:0030595	leukocyte che	236/18800	0.00026814	0.00221003	23
BP	GO:0007613	memory	122/18800	0.00026846	0.00221003	15
BP	GO:0051283	negative regu	122/18800	0.00026846	0.00221003	15
BP	GO:1903053	regulation of	50/18800	0.00026909	0.00221003	9
BP	GO:0001953	negative regu	40/18800	0.00027649	0.00225906	8
BP	GO:0010863	positive regul	40/18800	0.00027649	0.00225906	8
BP	GO:0007626	locomotory b	192/18800	0.00027707	0.00225906	20
BP	GO:0010817	regulation of	496/18800	0.000278	0.00226117	39
BP	GO:0030534	adult behavic	136/18800	0.00028334	0.00229905	16
BP	GO:0051235	maintenance	331/18800	0.00028932	0.00234194	29
BP	GO:0001764	neuron migra	164/18800	0.00029066	0.00234717	18
BP	GO:0051051	negative regu	464/18800	0.00030157	0.00242939	37
BP	GO:2000181	negative regu	151/18800	0.00031416	0.00251993	17
BP	GO:0060688	regulation of	51/18800	0.0003143	0.00251993	9
BP	GO:0035904	aorta develop	62/18800	0.00031739	0.00253862	10
BP	GO:0046683	response to c	124/18800	0.00032098	0.00255518	15
BP	GO:0051282	regulation of	124/18800	0.00032098	0.00255518	15
BP	GO:0014066	regulation of	111/18800	0.00032619	0.0025906	14
BP	GO:1902742	apoptotic prc	41/18800	0.00033057	0.00261299	8
BP	GO:1905314	semi-lunar va	41/18800	0.00033057	0.00261299	8
BP	GO:0007015	actin filamen	450/18800	0.00034055	0.00268557	36
BP	GO:0070371	ERK1 and ERK	335/18800	0.00035314	0.00277779	29
BP	GO:0014897	striated musc	99/18800	0.00035471	0.00277779	13
BP	GO:0050764	regulation of	99/18800	0.00035471	0.00277779	13
BP	GO:0032331	negative regu	23/18800	0.0003558	0.00277984	6
BP	GO:0060675	ureteric bud	63/18800	0.00036279	0.00282789	10
BP	GO:0070169	positive regul	52/18800	0.00036562	0.00284334	9
BP	GO:0003180	aortic valve n	32/18800	0.00037306	0.00288785	7
BP	GO:1901889	negative regu	32/18800	0.00037306	0.00288785	7
BP	GO:0010232	vascular trans	87/18800	0.00037478	0.00288789	12
BP	GO:0150104	transport acr	87/18800	0.00037478	0.00288789	12
BP	GO:0048588	development	227/18800	0.00039018	0.00297358	22

BP	GO:0007156	homophilic ce	168/18800	0.00039066	0.00297358	18
BP	GO:0015837	amine transp	100/18800	0.0003918	0.00297358	13
BP	GO:0018958	phenol-conta	113/18800	0.0003928	0.00297358	14
BP	GO:0014911	positive regul	42/18800	0.00039296	0.00297358	8
BP	GO:0030574	collagen cata	42/18800	0.00039296	0.00297358	8
BP	GO:1900274	regulation of	42/18800	0.00039296	0.00297358	8
BP	GO:1904706	negative regu	42/18800	0.00039296	0.00297358	8
BP	GO:0072171	mesonephric	64/18800	0.00041348	0.0031218	10
BP	GO:0008037	cell recogniti	228/18800	0.00041459	0.00312324	22
BP	GO:0110151	positive regul	53/18800	0.00042364	0.00318429	9
BP	GO:0014896	muscle hyper	101/18800	0.00043212	0.0032336	13
BP	GO:0099565	chemical syna	101/18800	0.00043212	0.0032336	13
BP	GO:0045986	negative regu	16/18800	0.00045053	0.00335641	5
BP	GO:0046068	cGMP metab	16/18800	0.00045053	0.00335641	5
BP	GO:0051208	sequestering	128/18800	0.00045292	0.00336681	15
BP	GO:0070482	response to c	324/18800	0.00045536	0.0033737	28
BP	GO:0033687	osteoblast pr	33/18800	0.0004559	0.0033737	7
BP	GO:0003081	regulation of	24/18800	0.00045707	0.0033737	6
BP	GO:0045926	negative regu	245/18800	0.00045786	0.0033737	23
BP	GO:0035107	appendage m	142/18800	0.00046223	0.00338468	16
BP	GO:0035108	limb morpho	142/18800	0.00046223	0.00338468	16
BP	GO:0001656	metanephros	89/18800	0.00046337	0.00338468	12
BP	GO:0051591	response to c	89/18800	0.00046337	0.00338468	12
BP	GO:1905330	regulation of	65/18800	0.00046991	0.00342503	10
BP	GO:0072028	nephron mor	77/18800	0.00047925	0.00348555	11
BP	GO:0030308	negative regu	186/18800	0.00049795	0.00361377	19
BP	GO:0030193	regulation of	66/18800	0.00053259	0.00385684	10
BP	GO:0017015	regulation of	130/18800	0.00053471	0.00386386	15
BP	GO:0007193	adenylate cyc	78/18800	0.0005364	0.00386777	11
BP	GO:0014065	phosphatidyli	144/18800	0.00054025	0.00388724	16
BP	GO:1904752	regulation of	44/18800	0.00054648	0.00392367	8
BP	GO:0031128	development	34/18800	0.00055277	0.00396039	7
BP	GO:0060541	respiratory sy	203/18800	0.00057179	0.00408792	20
BP	GO:0045932	negative regu	25/18800	0.00057946	0.00411659	6
BP	GO:0060343	trabecula for	25/18800	0.00057946	0.00411659	6
BP	GO:2000311	regulation of	25/18800	0.00057946	0.00411659	6
BP	GO:0032102	negative regu	429/18800	0.00058153	0.00412257	34
BP	GO:0055021	regulation of	67/18800	0.00060204	0.00424725	10
BP	GO:0043583	ear developm	219/18800	0.00060288	0.00424725	21
BP	GO:0051918	negative regu	10/18800	0.00060536	0.00424725	4
BP	GO:0060346	bone trabecu	10/18800	0.00060536	0.00424725	4
BP	GO:0043010	camera-type	330/18800	0.00060847	0.00424725	28
BP	GO:0001710	mesodermal	17/18800	0.00061551	0.00424725	5
BP	GO:0007158	neuron cell-c	17/18800	0.00061551	0.00424725	5

BP	GO:0051895	negative regu	17/18800	0.00061551	0.00424725	5
BP	GO:0055119	relaxation of	17/18800	0.00061551	0.00424725	5
BP	GO:0070593	dendrite self-	17/18800	0.00061551	0.00424725	5
BP	GO:0086103	G protein-cou	17/18800	0.00061551	0.00424725	5
BP	GO:0098962	regulation of	17/18800	0.00061551	0.00424725	5
BP	GO:0150118	negative regu	17/18800	0.00061551	0.00424725	5
BP	GO:0051952	regulation of	92/18800	0.00062895	0.00433108	12
BP	GO:1904738	vascular asso	45/18800	0.00063964	0.00439574	8
BP	GO:0003229	ventricular ca	56/18800	0.00064474	0.00442173	9
BP	GO:0050731	positive regul	190/18800	0.00064766	0.00443275	19
BP	GO:0050806	positive regul	161/18800	0.0006629	0.00451522	17
BP	GO:1905207	regulation of	35/18800	0.00066532	0.00451522	7
BP	GO:0030326	embryonic lin	119/18800	0.00066641	0.00451522	14
BP	GO:0035113	embryonic ap	119/18800	0.00066641	0.00451522	14
BP	GO:0051928	positive regul	119/18800	0.00066641	0.00451522	14
BP	GO:1903844	regulation of	133/18800	0.00068087	0.00460387	15
BP	GO:1902905	positive regul	176/18800	0.00068269	0.00460694	18
BP	GO:0032231	regulation of	106/18800	0.00069064	0.00465127	13
BP	GO:0060993	kidney morph	93/18800	0.00069412	0.00466537	12
BP	GO:0002026	regulation of	26/18800	0.00072583	0.00481131	6
BP	GO:0033622	integrin activ	26/18800	0.00072583	0.00481131	6
BP	GO:0036296	response to i	26/18800	0.00072583	0.00481131	6
BP	GO:0060259	regulation of	26/18800	0.00072583	0.00481131	6
BP	GO:0060314	regulation of	26/18800	0.00072583	0.00481131	6
BP	GO:0060561	apoptotic pro	26/18800	0.00072583	0.00481131	6
BP	GO:2000050	regulation of	26/18800	0.00072583	0.00481131	6
BP	GO:0060350	endochondra	57/18800	0.00073655	0.00487276	9
BP	GO:0098657	import into c	238/18800	0.00074206	0.00489955	22
BP	GO:0061383	trabecula mo	46/18800	0.0007452	0.00491071	8
BP	GO:0051962	positive regul	270/18800	0.00076246	0.00501073	24
BP	GO:0070527	platelet aggre	69/18800	0.00076346	0.00501073	10
BP	GO:0035249	synaptic trans	94/18800	0.00076485	0.00501073	12
BP	GO:0030100	regulation of	208/18800	0.00077778	0.00508556	20
BP	GO:0048675	axon extensio	121/18800	0.00078769	0.00514037	14
BP	GO:0046661	male sex diffe	164/18800	0.00081741	0.00532401	17
BP	GO:0043116	negative regu	18/18800	0.00082192	0.00532492	5
BP	GO:0046851	negative regu	18/18800	0.00082192	0.00532492	5
BP	GO:0007218	neuropeptide	108/18800	0.00082545	0.00532492	13
BP	GO:0071867	response to r	108/18800	0.00082545	0.00532492	13
BP	GO:0071869	response to c	108/18800	0.00082545	0.00532492	13
BP	GO:0045761	regulation of	58/18800	0.00083874	0.00536671	9
BP	GO:0060038	cardiac muscl	58/18800	0.00083874	0.00536671	9
BP	GO:0061005	cell differenti	58/18800	0.00083874	0.00536671	9
BP	GO:0014033	neural crest c	95/18800	0.0008415	0.00536671	12

BP	GO:0043502	regulation of	95/18800	0.0008415	0.00536671	12
BP	GO:0060079	excitatory po	95/18800	0.0008415	0.00536671	12
BP	GO:0010976	positive regul	150/18800	0.00084544	0.00538168	16
BP	GO:0014902	myotube diff	122/18800	0.00085499	0.00542206	14
BP	GO:0002548	monocyte ch	70/18800	0.00085662	0.00542206	10
BP	GO:1904888	cranial skelet	70/18800	0.00085662	0.00542206	10
BP	GO:0055010	ventricular ca	47/18800	0.00086433	0.00546064	8
BP	GO:0007162	negative regu	305/18800	0.00087602	0.0055241	26
BP	GO:0007416	synapse asse	180/18800	0.00088857	0.0055928	18
BP	GO:0007263	nitric oxide m	27/18800	0.00089922	0.00564924	6
BP	GO:0008360	regulation of	151/18800	0.00090841	0.00569636	16
BP	GO:0035581	sequestering	11/18800	0.00091861	0.00572835	4
BP	GO:0044557	relaxation of	11/18800	0.00091861	0.00572835	4
BP	GO:0070944	neutrophil-m	11/18800	0.00091861	0.00572835	4
BP	GO:0001708	cell fate speci	96/18800	0.00092445	0.00575407	12
BP	GO:0003176	aortic valve d	37/18800	0.0009445	0.00581249	7
BP	GO:0003203	endocardial c	37/18800	0.0009445	0.00581249	7
BP	GO:0045762	positive regu	37/18800	0.0009445	0.00581249	7
BP	GO:0090218	positive regul	37/18800	0.0009445	0.00581249	7
BP	GO:1901021	positive regul	37/18800	0.0009445	0.00581249	7
BP	GO:0018108	peptidyl-tyro	373/18800	0.0009463	0.00581249	30
BP	GO:0010721	negative regu	181/18800	0.00094764	0.00581249	18
BP	GO:0048017	inositol lipid-	181/18800	0.00094764	0.00581249	18
BP	GO:0014743	regulation of	71/18800	0.0009589	0.00582848	10
BP	GO:0050766	positive regul	71/18800	0.0009589	0.00582848	10
BP	GO:0050818	regulation of	71/18800	0.0009589	0.00582848	10
BP	GO:0060411	cardiac septu	71/18800	0.0009589	0.00582848	10
BP	GO:1904427	positive regul	71/18800	0.0009589	0.00582848	10
BP	GO:0043266	regulation of	110/18800	0.00098169	0.00595626	13
BP	GO:0030195	negative regu	48/18800	0.00099826	0.00602258	8
BP	GO:0043114	regulation of	48/18800	0.00099826	0.00602258	8
BP	GO:0048146	positive regul	48/18800	0.00099826	0.00602258	8
BP	GO:0042552	myelination	138/18800	0.00099978	0.00602258	15
BP	GO:0061053	somite devel	84/18800	0.00101172	0.00608363	11
BP	GO:0052548	regulation of	426/18800	0.00102541	0.00615498	33
BP	GO:0071692	protein locali	359/18800	0.00106744	0.00638919	29
BP	GO:0072078	nephron tubu	72/18800	0.00107099	0.00638919	10
BP	GO:0018212	peptidyl-tyro	376/18800	0.00107431	0.00638919	30
BP	GO:0006837	serotonin tra	19/18800	0.00107582	0.00638919	5
BP	GO:0061298	retina vascula	19/18800	0.00107582	0.00638919	5
BP	GO:0071625	vocalization b	19/18800	0.00107582	0.00638919	5
BP	GO:0050730	regulation of	261/18800	0.00109241	0.0064763	23
BP	GO:0014072	response to is	28/18800	0.0011028	0.00649219	6
BP	GO:0043278	response to r	28/18800	0.0011028	0.00649219	6

BP	GO:0090025	regulation of	28/18800	0.0011028	0.00649219	6
BP	GO:1903779	regulation of	28/18800	0.0011028	0.00649219	6
BP	GO:0009952	anterior/post	214/18800	0.00110669	0.00650372	20
BP	GO:0032965	regulation of	38/18800	0.0011149	0.0065292	7
BP	GO:1901385	regulation of	38/18800	0.0011149	0.0065292	7
BP	GO:0050886	endocrine pro	85/18800	0.00111738	0.00653232	11
BP	GO:0071695	anatomical st	246/18800	0.00114671	0.00668978	22
BP	GO:0046850	regulation of	49/18800	0.00114828	0.00668978	8
BP	GO:0007224	smoothened	140/18800	0.00115868	0.00669245	15
BP	GO:0007272	ensheathmer	140/18800	0.00115868	0.00669245	15
BP	GO:0008366	axon ensheat	140/18800	0.00115868	0.00669245	15
BP	GO:0008584	male gonad d	140/18800	0.00115868	0.00669245	15
BP	GO:0050921	positive regul	140/18800	0.00115868	0.00669245	15
BP	GO:0001707	mesoderm fo	73/18800	0.00119356	0.00688209	10
BP	GO:0045860	positive regul	396/18800	0.00121553	0.00697764	31
BP	GO:0032233	positive regul	61/18800	0.00121634	0.00697764	9
BP	GO:0099601	regulation of	61/18800	0.00121634	0.00697764	9
BP	GO:0007565	female pregn	185/18800	0.00121866	0.00697902	18
BP	GO:0034103	regulation of	86/18800	0.00123196	0.00704321	11
BP	GO:0046546	development	141/18800	0.00124578	0.00711019	15
BP	GO:0002040	sprouting ang	186/18800	0.00129587	0.00738356	18
BP	GO:0002691	regulation of	39/18800	0.00130851	0.00741789	7
BP	GO:0032570	response to p	39/18800	0.00130851	0.00741789	7
BP	GO:0051154	negative regu	39/18800	0.00130851	0.00741789	7
BP	GO:0031281	positive regul	50/18800	0.00131575	0.00743099	8
BP	GO:0060425	lung morphog	50/18800	0.00131575	0.00743099	8
BP	GO:0060420	regulation of	74/18800	0.00132733	0.00743099	10
BP	GO:0072088	nephron epit	74/18800	0.00132733	0.00743099	10
BP	GO:0007501	mesodermal	12/18800	0.00133068	0.00743099	4
BP	GO:0043568	positive regul	12/18800	0.00133068	0.00743099	4
BP	GO:0060394	negative regu	12/18800	0.00133068	0.00743099	4
BP	GO:0070943	neutrophil-m	12/18800	0.00133068	0.00743099	4
BP	GO:0086067	AV node cell	12/18800	0.00133068	0.00743099	4
BP	GO:0017145	stem cell divi	29/18800	0.00133992	0.00747021	6
BP	GO:0060393	regulation of	62/18800	0.001369	0.00761969	9
BP	GO:0003215	cardiac right	20/18800	0.0013835	0.0076624	5
BP	GO:0003228	atrial cardiac	20/18800	0.0013835	0.0076624	5
BP	GO:0044342	type B pancre	20/18800	0.0013835	0.0076624	5
BP	GO:0090596	sensory orgar	266/18800	0.00140558	0.00776394	23
BP	GO:0060560	development	234/18800	0.00140644	0.00776394	21
BP	GO:0000302	response to r	203/18800	0.00143113	0.00788728	19
BP	GO:0048332	mesoderm m	75/18800	0.00147305	0.00810508	10
BP	GO:0043277	apoptotic cell	51/18800	0.00150207	0.0082378	8
BP	GO:0051349	positive regul	51/18800	0.00150207	0.0082378	8

BP	GO:0099622	cardiac muscl	40/18800	0.00152742	0.00836323	7
BP	GO:0045453	bone resorpti	63/18800	0.0015367	0.00840036	9
BP	GO:0042476	odontogenes	130/18800	0.00158921	0.00867335	14
BP	GO:0045216	cell-cell junct	205/18800	0.00160425	0.00874126	19
BP	GO:0007190	activation of a	30/18800	0.00161405	0.00875217	6
BP	GO:0086011	membrane re	30/18800	0.00161405	0.00875217	6
BP	GO:0099623	regulation of	30/18800	0.00161405	0.00875217	6
BP	GO:0061333	renal tubule r	76/18800	0.0016315	0.00883258	10
BP	GO:1901652	response to p	491/18800	0.00163684	0.00884726	36
BP	GO:0031103	axon regener	52/18800	0.00170869	0.00919816	8
BP	GO:0050819	negative regu	52/18800	0.00170869	0.00919816	8
BP	GO:0060078	regulation of	131/18800	0.00170996	0.00919816	14
BP	GO:0060973	cell migration	21/18800	0.00175145	0.00939134	5
BP	GO:1905209	positive regul	21/18800	0.00175145	0.00939134	5
BP	GO:0106106	cold-induced	146/18800	0.0017683	0.00945163	15
BP	GO:0120161	regulation of	146/18800	0.0017683	0.00945163	15
BP	GO:0010463	mesenchyma	41/18800	0.00177383	0.00946616	7
BP	GO:0002028	regulation of	90/18800	0.00179105	0.00954294	11
BP	GO:0050772	positive regul	77/18800	0.00180348	0.00956374	10
BP	GO:0051149	positive regul	77/18800	0.00180348	0.00956374	10
BP	GO:0051339	regulation of	77/18800	0.00180348	0.00956374	10
BP	GO:0010002	cardioblast di	13/18800	0.00185629	0.00973649	4
BP	GO:0021819	layer formati	13/18800	0.00185629	0.00973649	4
BP	GO:0033689	negative regu	13/18800	0.00185629	0.00973649	4
BP	GO:0042659	regulation of	13/18800	0.00185629	0.00973649	4
BP	GO:0061430	bone trabecu	13/18800	0.00185629	0.00973649	4
BP	GO:1900115	extracellular r	13/18800	0.00185629	0.00973649	4
BP	GO:1900116	extracellular r	13/18800	0.00185629	0.00973649	4
BP	GO:0001659	temperature	177/18800	0.00189126	0.0098891	17
BP	GO:0048015	phosphatidyli	177/18800	0.00189126	0.0098891	17
BP	GO:0001756	somitogenesi	65/18800	0.00192148	0.01003154	9
BP	GO:0070528	protein kinas	31/18800	0.00192877	0.01005399	6
BP	GO:0010718	positive regul	53/18800	0.00193715	0.01006649	8
BP	GO:0060071	Wnt signaling	53/18800	0.00193715	0.01006649	8
BP	GO:0072080	nephron tubu	91/18800	0.00195909	0.01016483	11
BP	GO:0031279	regulation of	78/18800	0.00198981	0.01030837	10
BP	GO:0071868	cellular respo	105/18800	0.00202514	0.0104592	12
BP	GO:0071870	cellular respo	105/18800	0.00202514	0.0104592	12
BP	GO:0060412	ventricular se	42/18800	0.00205001	0.0105714	7
BP	GO:0009953	dorsal/ventra	92/18800	0.00213973	0.0110058	11
BP	GO:0060389	pathway-rest	66/18800	0.00214078	0.0110058	9
BP	GO:0007413	axonal fascicu	22/18800	0.00218629	0.01118534	5
BP	GO:0034104	negative regu	22/18800	0.00218629	0.01118534	5
BP	GO:0106030	neuron proje	22/18800	0.00218629	0.01118534	5

BP	GO:0071320	cellular respo	54/18800	0.00218899	0.01118534	8
BP	GO:0003279	cardiac septu	106/18800	0.0021955	0.01120162	12
BP	GO:0098703	calcium ion ir	32/18800	0.00228778	0.01165477	6
BP	GO:0007584	response to r	150/18800	0.00230788	0.01173939	15
BP	GO:0050767	regulation of	361/18800	0.00234024	0.01188604	28
BP	GO:0042593	glucose home	244/18800	0.00234738	0.01190436	21
BP	GO:0035019	somatic stem	43/18800	0.00235828	0.01192371	7
BP	GO:0045214	sarcomere or	43/18800	0.00235828	0.01192371	7
BP	GO:0030038	contractile ac	107/18800	0.0023774	0.01198439	12
BP	GO:0043149	stress fiber as	107/18800	0.0023774	0.01198439	12
BP	GO:0033500	carbohydrate	245/18800	0.00246549	0.01239313	21
BP	GO:0048008	platelet-deriv	55/18800	0.00246584	0.01239313	8
BP	GO:0001505	regulation of	213/18800	0.00248509	0.01246616	19
BP	GO:0010752	regulation of	14/18800	0.00250999	0.01246616	4
BP	GO:0038166	angiotensin-a	14/18800	0.00250999	0.01246616	4
BP	GO:0050930	induction of p	14/18800	0.00250999	0.01246616	4
BP	GO:0061043	regulation of	14/18800	0.00250999	0.01246616	4
BP	GO:0070942	neutrophil m	14/18800	0.00250999	0.01246616	4
BP	GO:1902043	positive regul	14/18800	0.00250999	0.01246616	4
BP	GO:1905065	positive regul	14/18800	0.00250999	0.01246616	4
BP	GO:0048709	oligodendroc	94/18800	0.00254156	0.01258585	11
BP	GO:0061326	renal tubule d	94/18800	0.00254156	0.01258585	11
BP	GO:0043542	endothelial c	279/18800	0.00260088	0.01286067	23
BP	GO:0042445	hormone met	230/18800	0.00261511	0.01291208	20
BP	GO:0010611	regulation of	68/18800	0.00263899	0.01297693	9
BP	GO:0043627	response to e	68/18800	0.00263899	0.01297693	9
BP	GO:0030512	negative regu	81/18800	0.00264366	0.01297693	10
BP	GO:0048144	fibroblast pro	81/18800	0.00264366	0.01297693	10
BP	GO:0003283	atrial septum	23/18800	0.00269476	0.0130494	5
BP	GO:0014821	phasic smoot	23/18800	0.00269476	0.0130494	5
BP	GO:0030194	positive regul	23/18800	0.00269476	0.0130494	5
BP	GO:0035988	chondrocyte	23/18800	0.00269476	0.0130494	5
BP	GO:1900048	positive regul	23/18800	0.00269476	0.0130494	5
BP	GO:0043552	positive regul	33/18800	0.00269487	0.0130494	6
BP	GO:0050901	leukocyte tet	33/18800	0.00269487	0.0130494	6
BP	GO:0070050	neuron cellul	33/18800	0.00269487	0.0130494	6
BP	GO:1902656	calcium ion ir	33/18800	0.00269487	0.0130494	6
BP	GO:0010828	positive regul	44/18800	0.00270105	0.0130494	7
BP	GO:0048286	lung alveolus	44/18800	0.00270105	0.0130494	7
BP	GO:1901379	regulation of	95/18800	0.00276419	0.01332208	11
BP	GO:0050433	regulation of	56/18800	0.00276936	0.01332208	8
BP	GO:0055078	sodium ion ho	56/18800	0.00276936	0.01332208	8
BP	GO:0034446	substrate adh	109/18800	0.00277807	0.01334491	12
BP	GO:0007266	Rho protein s	138/18800	0.00278663	0.01336695	14

BP	GO:0045834	positive regul	153/18800	0.00279663	0.01339584	15
BP	GO:0008589	regulation of	82/18800	0.00289625	0.0138533	10
BP	GO:0050795	regulation of	69/18800	0.00292032	0.01394864	9
BP	GO:0048639	positive regul	169/18800	0.00293	0.01397504	16
BP	GO:0009306	protein secre	350/18800	0.00298891	0.01423588	27
BP	GO:0048259	regulation of	110/18800	0.00299803	0.01425915	12
BP	GO:0003300	cardiac muscl	96/18800	0.00300227	0.01425919	11
BP	GO:0009612	response to r	201/18800	0.00305389	0.01448392	18
BP	GO:0050432	catecholamin	57/18800	0.00310125	0.01464662	8
BP	GO:0090175	regulation of	57/18800	0.00310125	0.01464662	8
BP	GO:0098900	regulation of	57/18800	0.00310125	0.01464662	8
BP	GO:0035592	establishmen	351/18800	0.00310837	0.01465971	27
BP	GO:0009954	proximal/dist	34/18800	0.00315389	0.01483282	6
BP	GO:0016486	peptide horm	34/18800	0.00315389	0.01483282	6
BP	GO:0043271	negative regu	155/18800	0.00316763	0.01487668	15
BP	GO:0002685	regulation of	218/18800	0.00321902	0.0150943	19
BP	GO:0051966	regulation of	70/18800	0.0032248	0.0150943	9
BP	GO:0001938	positive regul	111/18800	0.0032319	0.0150943	12
BP	GO:0031532	actin cytoskel	111/18800	0.0032319	0.0150943	12
BP	GO:0003181	atrioventricul	24/18800	0.00328363	0.01512531	5
BP	GO:0042730	fibrinolysis	24/18800	0.00328363	0.01512531	5
BP	GO:0048745	smooth musc	24/18800	0.00328363	0.01512531	5
BP	GO:0050820	positive regul	24/18800	0.00328363	0.01512531	5
BP	GO:0003214	cardiac left ve	15/18800	0.00330591	0.01512531	4
BP	GO:0010649	regulation of	15/18800	0.00330591	0.01512531	4
BP	GO:0010755	regulation of	15/18800	0.00330591	0.01512531	4
BP	GO:0010832	negative regu	15/18800	0.00330591	0.01512531	4
BP	GO:0032488	Cdc42 protein	15/18800	0.00330591	0.01512531	4
BP	GO:0032905	transforming	15/18800	0.00330591	0.01512531	4
BP	GO:0042415	norepinephri	15/18800	0.00330591	0.01512531	4
BP	GO:0060413	atrial septum	15/18800	0.00330591	0.01512531	4
BP	GO:0060572	morphogenes	15/18800	0.00330591	0.01512531	4
BP	GO:0060911	cardiac cell fa	15/18800	0.00330591	0.01512531	4
BP	GO:0099509	regulation of	15/18800	0.00330591	0.01512531	4
BP	GO:0051651	maintenance	219/18800	0.00338559	0.01544788	19
BP	GO:0071805	potassium ior	219/18800	0.00338559	0.01544788	19
BP	GO:0006584	catecholamin	58/18800	0.00346325	0.01573823	8
BP	GO:0009712	catechol-cont	58/18800	0.00346325	0.01573823	8
BP	GO:0048010	vascular endo	58/18800	0.00346325	0.01573823	8
BP	GO:0010830	regulation of	46/18800	0.00349999	0.0158624	7
BP	GO:0048701	embryonic cr	46/18800	0.00349999	0.0158624	7
BP	GO:0001666	response to h	286/18800	0.0035425	0.01603345	23
BP	GO:0070374	positive regul	220/18800	0.00355928	0.01608781	19
BP	GO:0010737	protein kinas	35/18800	0.00366876	0.01651614	6

BP	GO:0044319	wound healir	35/18800	0.00366876	0.01651614	6
BP	GO:0090505	epiboly invol\	35/18800	0.00366876	0.01651614	6
BP	GO:0010469	regulation of	173/18800	0.00369472	0.0166108	16
BP	GO:0044344	cellular respo	113/18800	0.00374386	0.01680927	12
BP	GO:0008543	fibroblast gro	85/18800	0.0037713	0.01690994	10
BP	GO:0001755	neural crest c	59/18800	0.00385714	0.01727183	8
BP	GO:0022604	regulation of	305/18800	0.00386423	0.01728058	24
BP	GO:0014855	striated musc	72/18800	0.0039084	0.01740874	9
BP	GO:0042246	tissue regene	72/18800	0.0039084	0.01740874	9
BP	GO:0051937	catecholamin	72/18800	0.0039084	0.01740874	9
BP	GO:0050769	positive regul	222/18800	0.00392893	0.01742964	19
BP	GO:0002438	acute inflamr	25/18800	0.00395967	0.01742964	5
BP	GO:0003148	outflow tract	25/18800	0.00395967	0.01742964	5
BP	GO:0006929	substrate-de	25/18800	0.00395967	0.01742964	5
BP	GO:0032104	regulation of	25/18800	0.00395967	0.01742964	5
BP	GO:0032107	regulation of	25/18800	0.00395967	0.01742964	5
BP	GO:0032967	positive regul	25/18800	0.00395967	0.01742964	5
BP	GO:0060074	synapse matu	25/18800	0.00395967	0.01742964	5
BP	GO:1904753	negative regu	25/18800	0.00395967	0.01742964	5
BP	GO:0001894	tissue homeo	272/18800	0.00400118	0.01758935	22
BP	GO:1990845	adaptive ther	159/18800	0.00403132	0.01769875	15
BP	GO:0045927	positive regul	256/18800	0.00413056	0.01811081	21
BP	GO:0035909	aorta morpho	36/18800	0.00424346	0.01847587	6
BP	GO:0090504	epiboly	36/18800	0.00424346	0.01847587	6
BP	GO:0140448	signaling rece	36/18800	0.00424346	0.01847587	6
BP	GO:0008228	opsonization	16/18800	0.00425771	0.01847587	4
BP	GO:0072109	glomerular m	16/18800	0.00425771	0.01847587	4
BP	GO:0072224	metanephric	16/18800	0.00425771	0.01847587	4
BP	GO:1903236	regulation of	16/18800	0.00425771	0.01847587	4
BP	GO:2000095	regulation of	16/18800	0.00425771	0.01847587	4
BP	GO:0046888	negative regu	60/18800	0.00428474	0.01852126	8
BP	GO:0055008	cardiac muscl	60/18800	0.00428474	0.01852126	8
BP	GO:0003281	ventricular se	73/18800	0.00429017	0.01852126	9
BP	GO:0019226	transmission	73/18800	0.00429017	0.01852126	9
BP	GO:0046620	regulation of	101/18800	0.00445179	0.0191379	11
BP	GO:0050829	defense resp	87/18800	0.00446249	0.0191379	10
BP	GO:0006953	acute-phase	48/18800	0.00446711	0.0191379	7
BP	GO:0031641	regulation of	48/18800	0.00446711	0.0191379	7
BP	GO:0035850	epithelial cell	48/18800	0.00446711	0.0191379	7
BP	GO:0060976	coronary vas	48/18800	0.00446711	0.0191379	7
BP	GO:1901653	cellular respo	361/18800	0.00454247	0.01943605	27
BP	GO:0050807	regulation of	209/18800	0.00461096	0.01970404	18
BP	GO:0006956	complement	131/18800	0.00468483	0.01999436	13
BP	GO:0035924	cellular respo	74/18800	0.00470041	0.02003324	9

BP	GO:0010714	positive regul	26/18800	0.00472964	0.02003324	5
BP	GO:0014829	vascular asso	26/18800	0.00472964	0.02003324	5
BP	GO:0030318	melanocyte d	26/18800	0.00472964	0.02003324	5
BP	GO:0043931	ossification ir	26/18800	0.00472964	0.02003324	5
BP	GO:1904385	cellular respo	26/18800	0.00472964	0.02003324	5
BP	GO:0021885	forebrain cell	61/18800	0.0047479	0.02006013	8
BP	GO:0032387	negative regu	61/18800	0.0047479	0.02006013	8
BP	GO:0014031	mesenchyma	88/18800	0.00484356	0.02040972	10
BP	GO:0048864	stem cell dev	88/18800	0.00484356	0.02040972	10
BP	GO:0006813	potassium ior	243/18800	0.00486873	0.02040972	20
BP	GO:0003230	cardiac atriur	37/18800	0.00488196	0.02040972	6
BP	GO:0014741	negative regu	37/18800	0.00488196	0.02040972	6
BP	GO:0033144	negative regu	37/18800	0.00488196	0.02040972	6
BP	GO:0048846	axon extensic	37/18800	0.00488196	0.02040972	6
BP	GO:1902284	neuron proje	37/18800	0.00488196	0.02040972	6
BP	GO:0001935	endothelial c	194/18800	0.00488519	0.02040972	17
BP	GO:0050873	brown fat cel	49/18800	0.00502024	0.02094796	7
BP	GO:0050680	negative regu	163/18800	0.0050783	0.02116398	15
BP	GO:0051963	regulation of	103/18800	0.00516775	0.02151014	11
BP	GO:0022617	extracellular	62/18800	0.00524848	0.02181921	8
BP	GO:0003198	epithelial to r	17/18800	0.00537839	0.02224939	4
BP	GO:0030949	positive regul	17/18800	0.00537839	0.02224939	4
BP	GO:0038065	collagen-activ	17/18800	0.00537839	0.02224939	4
BP	GO:0090136	epithelial cell	17/18800	0.00537839	0.02224939	4
BP	GO:0001990	regulation of	38/18800	0.00558829	0.02299742	6
BP	GO:0035886	vascular asso	38/18800	0.00558829	0.02299742	6
BP	GO:0040036	regulation of	38/18800	0.00558829	0.02299742	6
BP	GO:0003171	atrioventricul	27/18800	0.00560018	0.02299742	5
BP	GO:0071880	adenylate cyc	27/18800	0.00560018	0.02299742	5
BP	GO:1903055	positive regul	27/18800	0.00560018	0.02299742	5
BP	GO:0019933	cAMP-mediat	50/18800	0.0056233	0.02300816	7
BP	GO:0038084	vascular endc	50/18800	0.0056233	0.02300816	7
BP	GO:1902041	regulation of	50/18800	0.0056233	0.02300816	7
BP	GO:0071774	response to f	119/18800	0.00568159	0.02321844	12
BP	GO:0043434	response to p	404/18800	0.00592718	0.02419273	29
BP	GO:0036293	response to c	299/18800	0.00605229	0.02467346	23
BP	GO:0090100	positive regul	120/18800	0.00607047	0.02471765	12
BP	GO:0015850	organic hydrc	248/18800	0.00608185	0.02473408	20
BP	GO:0060021	roof of moutl	91/18800	0.00614167	0.02494725	10
BP	GO:0050803	regulation of	215/18800	0.00616481	0.02501109	18
BP	GO:0043268	positive regul	51/18800	0.00627895	0.02539031	7
BP	GO:0051496	positive regul	51/18800	0.00627895	0.02539031	7
BP	GO:0071674	mononuclear	199/18800	0.00628091	0.02539031	17
BP	GO:0032103	positive regul	442/18800	0.00631803	0.02550975	31

BP	GO:0043267	negative regu	39/18800	0.00636643	0.02559481	6
BP	GO:0098815	modulation o	39/18800	0.00636643	0.02559481	6
BP	GO:1901020	negative regu	39/18800	0.00636643	0.02559481	6
BP	GO:0009620	response to f	64/18800	0.0063695	0.02559481	8
BP	GO:0007631	feeding beha	106/18800	0.00640901	0.02572288	11
BP	GO:0009991	response to e	479/18800	0.00642851	0.02577043	33
BP	GO:0003272	endocardial c	28/18800	0.00657788	0.02633785	5
BP	GO:0051492	regulation of	92/18800	0.00662971	0.02651388	10
BP	GO:0007422	peripheral ne	78/18800	0.0066541	0.02654835	9
BP	GO:0010827	regulation of	78/18800	0.0066541	0.02654835	9
BP	GO:0003128	heart field sp	18/18800	0.00668023	0.02655821	4
BP	GO:0031643	positive regul	18/18800	0.00668023	0.02655821	4
BP	GO:0097094	craniofacial s	18/18800	0.00668023	0.02655821	4
BP	GO:0035176	social behavi	52/18800	0.00698988	0.02775648	7
BP	GO:1902904	negative regu	169/18800	0.00705175	0.02796917	15
BP	GO:0034109	homotypic ce	93/18800	0.00714731	0.02831487	10
BP	GO:0007212	dopamine rec	40/18800	0.00722037	0.02849993	6
BP	GO:0072210	metanephric	40/18800	0.00722037	0.02849993	6
BP	GO:1903115	regulation of	40/18800	0.00722037	0.02849993	6
BP	GO:0048145	regulation of	79/18800	0.00722788	0.02849993	9
BP	GO:0042471	ear morphog	123/18800	0.00736475	0.02900563	12
BP	GO:0050770	regulation of	154/18800	0.00738973	0.02907004	14
BP	GO:0003209	cardiac atriur	29/18800	0.00766912	0.02978632	5
BP	GO:0010667	negative regu	29/18800	0.00766912	0.02978632	5
BP	GO:0048265	response to p	29/18800	0.00766912	0.02978632	5
BP	GO:0051481	negative regu	29/18800	0.00766912	0.02978632	5
BP	GO:0051968	positive regul	29/18800	0.00766912	0.02978632	5
BP	GO:0060037	pharyngeal sy	29/18800	0.00766912	0.02978632	5
BP	GO:0060441	epithelial tub	29/18800	0.00766912	0.02978632	5
BP	GO:0061082	myeloid leuk	29/18800	0.00766912	0.02978632	5
BP	GO:0070977	bone matura	29/18800	0.00766912	0.02978632	5
BP	GO:0099068	postsynapse a	29/18800	0.00766912	0.02978632	5
BP	GO:0099625	ventricular ca	29/18800	0.00766912	0.02978632	5
BP	GO:1901655	cellular respo	94/18800	0.00769562	0.02985481	10
BP	GO:0050832	defense resp	53/18800	0.00775877	0.03002064	7
BP	GO:0001542	ovulation fro	10/18800	0.00782751	0.03002064	3
BP	GO:0008343	adult feeding	10/18800	0.00782751	0.03002064	3
BP	GO:0010749	regulation of	10/18800	0.00782751	0.03002064	3
BP	GO:0060920	cardiac pacer	10/18800	0.00782751	0.03002064	3
BP	GO:0061626	pharyngeal ar	10/18800	0.00782751	0.03002064	3
BP	GO:0098903	regulation of	10/18800	0.00782751	0.03002064	3
BP	GO:1901387	positive regul	10/18800	0.00782751	0.03002064	3
BP	GO:1904338	regulation of	10/18800	0.00782751	0.03002064	3
BP	GO:2000425	regulation of	10/18800	0.00782751	0.03002064	3

BP	GO:0021983	pituitary glan	41/18800	0.00815407	0.03113956	6
BP	GO:0002523	leukocyte mig	19/18800	0.00817474	0.03113956	4
BP	GO:0010738	regulation of	19/18800	0.00817474	0.03113956	4
BP	GO:0032332	positive regul	19/18800	0.00817474	0.03113956	4
BP	GO:0051152	positive regul	19/18800	0.00817474	0.03113956	4
BP	GO:0060977	coronary vasc	19/18800	0.00817474	0.03113956	4
BP	GO:1990138	neuron proje	172/18800	0.00824627	0.03137651	15
BP	GO:1901016	regulation of	67/18800	0.00837958	0.03184779	8
BP	GO:0045445	myoblast diff	81/18800	0.00848811	0.03222389	9
BP	GO:0051155	positive regul	54/18800	0.00858831	0.03253095	7
BP	GO:0051703	biological pro	54/18800	0.00858831	0.03253095	7
BP	GO:1903532	positive regul	274/18800	0.00880739	0.03332331	21
BP	GO:0031069	hair follicle m	30/18800	0.00888013	0.03348564	5
BP	GO:0034368	protein-lipid	30/18800	0.00888013	0.03348564	5
BP	GO:0034369	plasma lipopr	30/18800	0.00888013	0.03348564	5
BP	GO:0045598	regulation of	142/18800	0.00910634	0.03430021	13
BP	GO:0051148	negative regu	68/18800	0.00914499	0.03440733	8
BP	GO:0014003	oligodendroc	42/18800	0.00917141	0.03446821	6
BP	GO:0006909	phagocytosis	310/18800	0.00918602	0.03448464	23
BP	GO:0043279	response to a	97/18800	0.00953639	0.03576005	10
BP	GO:0071902	positive regul	208/18800	0.00959751	0.03594921	17
BP	GO:0035313	wound healin	20/18800	0.00987255	0.03674401	4
BP	GO:0043586	tongue devel	20/18800	0.00987255	0.03674401	4
BP	GO:0061050	regulation of	20/18800	0.00987255	0.03674401	4
BP	GO:2000479	regulation of	20/18800	0.00987255	0.03674401	4
BP	GO:2000738	positive regul	20/18800	0.00987255	0.03674401	4
BP	GO:0048839	inner ear dev	192/18800	0.00987517	0.03674401	16
BP	GO:0014032	neural crest c	83/18800	0.00990844	0.0367865	9
BP	GO:0032413	negative regu	83/18800	0.00990844	0.0367865	9
BP	GO:0043550	regulation of	69/18800	0.00996135	0.0369422	8
BP	GO:0016485	protein proce	243/18800	0.01009723	0.03728206	19
BP	GO:0051592	response to c	144/18800	0.01017995	0.03728206	13
BP	GO:0002526	acute inflamr	113/18800	0.01021363	0.03728206	11
BP	GO:0031623	receptor inte	113/18800	0.01021363	0.03728206	11
BP	GO:1904659	glucose trans	113/18800	0.01021363	0.03728206	11
BP	GO:0010664	negative regu	31/18800	0.01021693	0.03728206	5
BP	GO:0033137	negative regu	31/18800	0.01021693	0.03728206	5
BP	GO:0060055	angiogenesis	31/18800	0.01021693	0.03728206	5
BP	GO:0060351	cartilage deve	31/18800	0.01021693	0.03728206	5
BP	GO:0061036	positive regul	31/18800	0.01021693	0.03728206	5
BP	GO:0071295	cellular respo	31/18800	0.01021693	0.03728206	5
BP	GO:0098868	bone growth	31/18800	0.01021693	0.03728206	5
BP	GO:0120162	positive regul	98/18800	0.01021917	0.03728206	10
BP	GO:1904063	negative regu	98/18800	0.01021917	0.03728206	10

BP	GO:0016339	calcium-depe	43/18800	0.01027624	0.03728206	6
BP	GO:0051150	regulation of	43/18800	0.01027624	0.03728206	6
BP	GO:0071526	semaphorin-p	43/18800	0.01027624	0.03728206	6
BP	GO:0006182	cGMP biosyn	11/18800	0.01041835	0.03728206	3
BP	GO:0010739	positive regul	11/18800	0.01041835	0.03728206	3
BP	GO:0032429	regulation of	11/18800	0.01041835	0.03728206	3
BP	GO:0048103	somatic stem	11/18800	0.01041835	0.03728206	3
BP	GO:0051610	serotonin upt	11/18800	0.01041835	0.03728206	3
BP	GO:0060601	lateral sprout	11/18800	0.01041835	0.03728206	3
BP	GO:0061303	cornea devel	11/18800	0.01041835	0.03728206	3
BP	GO:0086016	AV node cell a	11/18800	0.01041835	0.03728206	3
BP	GO:0086027	AV node cell t	11/18800	0.01041835	0.03728206	3
BP	GO:0086070	SA node cell t	11/18800	0.01041835	0.03728206	3
BP	GO:0097084	vascular asso	11/18800	0.01041835	0.03728206	3
BP	GO:0097104	postsynaptic	11/18800	0.01041835	0.03728206	3
BP	GO:0098883	synapse prun	11/18800	0.01041835	0.03728206	3
BP	GO:1903238	positive regul	11/18800	0.01041835	0.03728206	3
BP	GO:1905809	negative regu	11/18800	0.01041835	0.03728206	3
BP	GO:2000253	positive regul	11/18800	0.01041835	0.03728206	3
BP	GO:0045806	negative regu	56/18800	0.01044001	0.0373199	7
BP	GO:0001101	response to a	129/18800	0.01059671	0.03783991	12
BP	GO:0043200	response to a	114/18800	0.01087474	0.03879157	11
BP	GO:0051588	regulation of	99/18800	0.01093852	0.03897781	10
BP	GO:0001936	regulation of	178/18800	0.01111553	0.03956669	15
BP	GO:0051495	positive regul	195/18800	0.01134886	0.04035459	16
BP	GO:0006898	receptor-med	246/18800	0.0114071	0.04051888	19
BP	GO:0043551	regulation of	57/18800	0.01146743	0.04057935	7
BP	GO:0021795	cerebral corte	44/18800	0.01147232	0.04057935	6
BP	GO:0060443	mammary gla	44/18800	0.01147232	0.04057935	6
BP	GO:1901381	positive regul	44/18800	0.01147232	0.04057935	6
BP	GO:0046879	hormone secr	281/18800	0.01151407	0.04067151	21
BP	GO:0002683	negative regu	425/18800	0.01152254	0.04067151	29
BP	GO:0021782	glial cell deve	115/18800	0.01156812	0.04077584	11
BP	GO:0002063	chondrocyte	32/18800	0.01168529	0.04077584	5
BP	GO:0010743	regulation of	32/18800	0.01168529	0.04077584	5
BP	GO:0030947	regulation of	32/18800	0.01168529	0.04077584	5
BP	GO:0034367	protein-conta	32/18800	0.01168529	0.04077584	5
BP	GO:0060795	cell fate comr	32/18800	0.01168529	0.04077584	5
BP	GO:0060914	heart formati	32/18800	0.01168529	0.04077584	5
BP	GO:0070570	regulation of	32/18800	0.01168529	0.04077584	5
BP	GO:0071711	basement me	32/18800	0.01168529	0.04077584	5
BP	GO:0090183	regulation of	32/18800	0.01168529	0.04077584	5
BP	GO:1901380	negative regu	32/18800	0.01168529	0.04077584	5
BP	GO:0031290	retinal gangli	21/18800	0.0117834	0.04094846	4

BP	GO:0071636	positive regul	21/18800	0.0117834	0.04094846	4
BP	GO:0090189	regulation of	21/18800	0.0117834	0.04094846	4
BP	GO:0150146	cell junction (	21/18800	0.0117834	0.04094846	4
BP	GO:0099173	postsynapse (	163/18800	0.01188161	0.04124716	14
BP	GO:0051047	positive regul	300/18800	0.01210941	0.04199469	22
BP	GO:0031667	response to r	446/18800	0.01240944	0.0429909	30
BP	GO:0110020	regulation of	101/18800	0.01249164	0.0432312	10
BP	GO:0002090	regulation of	58/18800	0.01256605	0.04335502	7
BP	GO:0022029	telencephalo	58/18800	0.01256605	0.04335502	7
BP	GO:0042733	embryonic di	58/18800	0.01256605	0.04335502	7
BP	GO:0001736	establishmen	72/18800	0.0127354	0.04372211	8
BP	GO:0007164	establishmen	72/18800	0.0127354	0.04372211	8
BP	GO:0033143	regulation of	72/18800	0.0127354	0.04372211	8
BP	GO:0042551	neuron matu	45/18800	0.01276333	0.04372211	6
BP	GO:1900271	regulation of	45/18800	0.01276333	0.04372211	6
BP	GO:1903170	negative regu	45/18800	0.01276333	0.04372211	6
BP	GO:2000273	positive regul	45/18800	0.01276333	0.04372211	6
BP	GO:0006814	sodium ion tr	249/18800	0.01284878	0.0439701	19
BP	GO:0008645	hexose transı	117/18800	0.01305555	0.04463234	11
BP	GO:0051494	negative regu	165/18800	0.01312061	0.04480926	14
BP	GO:0010765	positive regul	33/18800	0.01329073	0.04505764	5
BP	GO:0021799	cerebral corte	33/18800	0.01329073	0.04505764	5
BP	GO:0030511	positive regul	33/18800	0.01329073	0.04505764	5
BP	GO:0048799	animal organ	33/18800	0.01329073	0.04505764	5
BP	GO:0071875	adrenergic re	33/18800	0.01329073	0.04505764	5
BP	GO:0099084	postsynaptic	33/18800	0.01329073	0.04505764	5
BP	GO:1903846	positive regul	33/18800	0.01329073	0.04505764	5
BP	GO:0006910	phagocytosis,	102/18800	0.01332778	0.04505764	10
BP	GO:0001867	complement	12/18800	0.01344757	0.04505764	3
BP	GO:0010642	negative regu	12/18800	0.01344757	0.04505764	3
BP	GO:0032908	regulation of	12/18800	0.01344757	0.04505764	3
BP	GO:0033629	negative regu	12/18800	0.01344757	0.04505764	3
BP	GO:0035112	genitalia mor	12/18800	0.01344757	0.04505764	3
BP	GO:0038063	collagen-activ	12/18800	0.01344757	0.04505764	3
BP	GO:0051001	negative regu	12/18800	0.01344757	0.04505764	3
BP	GO:0061469	regulation of	12/18800	0.01344757	0.04505764	3
BP	GO:0072537	fibroblast act	12/18800	0.01344757	0.04505764	3
BP	GO:1900272	negative regu	12/18800	0.01344757	0.04505764	3
BP	GO:2000035	regulation of	12/18800	0.01344757	0.04505764	3
BP	GO:0098659	inorganic cati	118/18800	0.01385154	0.04585187	11
BP	GO:0099587	inorganic ion	118/18800	0.01385154	0.04585187	11
BP	GO:0006883	cellular sodiu	22/18800	0.01391609	0.04585187	4
BP	GO:0007620	copulation	22/18800	0.01391609	0.04585187	4
BP	GO:0009190	cyclic nucleot	22/18800	0.01391609	0.04585187	4

BP	GO:0032288	myelin assem	22/18800	0.01391609	0.04585187	4
BP	GO:0035929	steroid horm	22/18800	0.01391609	0.04585187	4
BP	GO:0046058	cAMP metab	22/18800	0.01391609	0.04585187	4
BP	GO:0046597	negative regu	22/18800	0.01391609	0.04585187	4
BP	GO:0048485	sympathetic	22/18800	0.01391609	0.04585187	4
BP	GO:0052652	cyclic purine	22/18800	0.01391609	0.04585187	4
BP	GO:0061042	vascular wou	22/18800	0.01391609	0.04585187	4
BP	GO:0061318	renal filtratio	22/18800	0.01391609	0.04585187	4
BP	GO:0071305	cellular respo	22/18800	0.01391609	0.04585187	4
BP	GO:0072112	glomerular vi	22/18800	0.01391609	0.04585187	4
BP	GO:0072574	hepatocyte p	22/18800	0.01391609	0.04585187	4
BP	GO:0072575	epithelial cell	22/18800	0.01391609	0.04585187	4
BP	GO:0043405	regulation of	183/18800	0.01405992	0.04628046	15
BP	GO:0001974	blood vessel	46/18800	0.01415283	0.04649541	6
BP	GO:1904707	positive regul	46/18800	0.01415283	0.04649541	6
BP	GO:0035282	segmentation	103/18800	0.0142052	0.04662197	10
BP	GO:1901879	regulation of	88/18800	0.01423778	0.04668341	9
BP	GO:0023061	signal release	451/18800	0.01430186	0.04684789	30
BP	GO:0006979	response to c	433/18800	0.01455283	0.04762364	29
BP	GO:0015749	monosacchar	119/18800	0.01468367	0.04800516	11
BP	GO:0007435	salivary gland	34/18800	0.01503846	0.04892756	5
BP	GO:0048536	spleen develo	34/18800	0.01503846	0.04892756	5
BP	GO:0048730	epidermis mo	34/18800	0.01503846	0.04892756	5
BP	GO:0086004	regulation of	34/18800	0.01503846	0.04892756	5
BP	GO:1900181	negative regu	34/18800	0.01503846	0.04892756	5
BP	GO:0002444	myeloid leuko	104/18800	0.01512508	0.04911449	10
BP	GO:0034766	negative regu	104/18800	0.01512508	0.04911449	10
BP	GO:0051781	positive regul	89/18800	0.01524894	0.04946899	9
CC	GO:0062023	collagen-cont	429/19594	8.74E-62	2.89E-59	118
CC	GO:0043292	contractile fib	238/19594	5.14E-20	8.50E-18	49
CC	GO:0030016	myofibril	228/19594	2.85E-19	3.14E-17	47
CC	GO:0030017	sarcomere	209/19594	1.05E-17	8.68E-16	43
CC	GO:0030018	Z disc	127/19594	2.65E-15	1.75E-13	31
CC	GO:0031674	I band	139/19594	5.68E-15	3.13E-13	32
CC	GO:0042383	sarcolemma	131/19594	6.70E-15	3.16E-13	31
CC	GO:0005581	collagen trim	86/19594	1.67E-14	6.90E-13	25
CC	GO:0043025	neuronal cell	482/19594	6.49E-14	2.38E-12	61
CC	GO:0005788	endoplasmic	311/19594	3.40E-13	1.12E-11	46
CC	GO:0005604	basement me	95/19594	8.93E-11	2.68E-09	22
CC	GO:0031091	platelet alpha	91/19594	1.71E-09	4.72E-08	20
CC	GO:0098636	protein comp	43/19594	1.94E-09	4.86E-08	14
CC	GO:0030055	cell-substrate	428/19594	2.06E-09	4.86E-08	48
CC	GO:0032432	actin filamen	78/19594	4.86E-09	1.07E-07	18
CC	GO:0098644	complex of co	22/19594	9.42E-09	1.95E-07	10

CC	GO:0072562	blood microp	147/19594	2.25E-08	4.38E-07	24
CC	GO:0005925	focal adhesio	419/19594	2.48E-08	4.55E-07	45
CC	GO:0001725	stress fiber	69/19594	3.23E-08	5.33E-07	16
CC	GO:0097517	contractile ac	69/19594	3.23E-08	5.33E-07	16
CC	GO:0043204	perikaryon	153/19594	4.98E-08	7.84E-07	24
CC	GO:0005901	caveola	82/19594	7.11E-08	1.07E-06	17
CC	GO:0042641	actomyosin	77/19594	1.66E-07	2.39E-06	16
CC	GO:0030315	T-tubule	51/19594	1.91E-07	2.63E-06	13
CC	GO:0044853	plasma meml	113/19594	4.05E-07	5.36E-06	19
CC	GO:0031093	platelet alpha	67/19594	9.12E-07	1.16E-05	14
CC	GO:0005911	cell-cell junct	497/19594	1.33E-06	1.62E-05	46
CC	GO:0034774	secretory gra	322/19594	1.86E-06	2.20E-05	34
CC	GO:0060205	cytoplasmic v	325/19594	2.29E-06	2.60E-05	34
CC	GO:0043034	costamere	16/19594	2.36E-06	2.60E-05	7
CC	GO:0031983	vesicle lumen	327/19594	2.63E-06	2.80E-05	34
CC	GO:0097060	synaptic mem	373/19594	2.94E-06	3.04E-05	37
CC	GO:0015629	actin cytoskel	499/19594	3.45E-06	3.46E-05	45
CC	GO:0008305	integrin comp	31/19594	4.61E-06	4.44E-05	9
CC	GO:0001527	microfibril	12/19594	4.97E-06	4.44E-05	6
CC	GO:0005583	fibrillar collag	12/19594	4.97E-06	4.44E-05	6
CC	GO:0098643	banded collag	12/19594	4.97E-06	4.44E-05	6
CC	GO:0016528	sarcoplasm	78/19594	6.11E-06	5.32E-05	14
CC	GO:0045211	postsynaptic	271/19594	8.09E-06	6.86E-05	29
CC	GO:0098984	neuron to ne	347/19594	9.58E-06	7.91E-05	34
CC	GO:0016529	sarcoplasmic	71/19594	1.04E-05	8.40E-05	13
CC	GO:0032279	asymmetric s	323/19594	1.39E-05	0.00010945	32
CC	GO:0005775	vacuolar lum	174/19594	2.38E-05	0.00018308	21
CC	GO:0014069	postsynaptic	318/19594	2.58E-05	0.00019418	31
CC	GO:0099572	postsynaptic	341/19594	4.08E-05	0.00029933	32
CC	GO:0045121	membrane ra	326/19594	4.17E-05	0.00029933	31
CC	GO:0098857	membrane m	327/19594	4.42E-05	0.00031052	31
CC	GO:0044306	neuron proje	129/19594	4.57E-05	0.00031436	17
CC	GO:0009897	external side	455/19594	4.77E-05	0.0003218	39
CC	GO:0150034	distal axon	270/19594	5.44E-05	0.00035726	27
CC	GO:0016010	dystrophin-as	17/19594	5.51E-05	0.00035726	6
CC	GO:0098793	presynapse	492/19594	5.81E-05	0.00036955	41
CC	GO:0043197	dendritic spir	172/19594	6.30E-05	0.00039262	20
CC	GO:0044309	neuron spine	173/19594	6.83E-05	0.00041827	20
CC	GO:0043679	axon terminu	113/19594	0.00011578	0.00069579	15
CC	GO:0098992	neuronal den	13/19594	0.0001493	0.00088119	5
CC	GO:0090665	glycoprotein	21/19594	0.00020809	0.00120666	6
CC	GO:0014704	intercalated	49/19594	0.00023257	0.00132461	9
CC	GO:0044291	cell-cell conta	71/19594	0.00023645	0.00132461	11
CC	GO:0034703	cation chann	221/19594	0.00027591	0.00151991	22

CC	GO:0005884	actin filamen	113/19594	0.00040036	0.00216932	14
CC	GO:0036379	myofilament	25/19594	0.00058501	0.00306972	6
CC	GO:0005796	Golgi lumen	104/19594	0.00058511	0.00306972	13
CC	GO:0034704	calcium chan	68/19594	0.00068858	0.00355616	10
CC	GO:0043198	dendritic sha	36/19594	0.00080384	0.00408754	7
CC	GO:0031527	filopodium m	18/19594	0.00082859	0.00408761	5
CC	GO:0090533	cation-transp	18/19594	0.00082859	0.00408761	5
CC	GO:0005614	interstitial m	11/19594	0.00092473	0.00442967	4
CC	GO:1990454	L-type voltag	11/19594	0.00092473	0.00442967	4
CC	GO:0043202	lysosomal lu	97/19594	0.00103057	0.00486617	12
CC	GO:0034702	ion channel c	294/19594	0.00116126	0.00540599	25
CC	GO:0031941	filamentous a	29/19594	0.00135236	0.00620819	6
CC	GO:0098839	postsynaptic	89/19594	0.00165914	0.00751217	11
CC	GO:0099240	intrinsic com	160/19594	0.00170984	0.00755307	16
CC	GO:0098978	glutamatergi	319/19594	0.00171387	0.00755307	26
CC	GO:0005865	striated musc	21/19594	0.00176528	0.00767725	5
CC	GO:0031258	lamellipodiur	22/19594	0.00220341	0.00945824	5
CC	GO:0098936	intrinsic com	123/19594	0.00276086	0.01169919	13
CC	GO:0031225	anchored cor	169/19594	0.0029849	0.01248844	16
CC	GO:0098533	ATPase depe	24/19594	0.00330888	0.0136709	5
CC	GO:0005869	dynactin com	16/19594	0.00428464	0.01748378	4
CC	GO:1902495	transmembra	377/19594	0.00440908	0.01777214	28
CC	GO:0099634	postsynaptic	116/19594	0.0047002	0.01844597	12
CC	GO:0031045	dense core gr	26/19594	0.00476535	0.01844597	5
CC	GO:0034706	sodium chan	26/19594	0.00476535	0.01844597	5
CC	GO:0044304	main axon	61/19594	0.00479948	0.01844597	8
CC	GO:1990351	transporter c	399/19594	0.00513671	0.01951516	29
CC	GO:0031092	platelet alpha	17/19594	0.00541205	0.02032757	4
CC	GO:0099699	integral comp	149/19594	0.00564382	0.0209599	14
CC	GO:0030175	filopodium	105/19594	0.00605236	0.02222736	11
CC	GO:0032280	symmetric sy	10/19594	0.00786539	0.02856832	3
CC	GO:0033017	sarcoplasmic	41/19594	0.00822274	0.02923152	6
CC	GO:0060077	inhibitory syn	19/19594	0.00822486	0.02923152	4
CC	GO:0031594	neuromuscul	67/19594	0.00846686	0.0297715	8
CC	GO:1904090	peptidase inh	11/19594	0.01046813	0.03642098	3
CC	GO:0005891	voltage-gatec	44/19594	0.01156654	0.0398234	6
CC	GO:0099055	integral comp	118/19594	0.01402442	0.04778805	11
MF	GO:0005201	extracellular	172/18410	5.37E-37	3.33E-34	59
MF	GO:0008201	heparin bindi	168/18410	3.28E-27	1.02E-24	49
MF	GO:0005539	glycosaminog	234/18410	5.34E-27	1.11E-24	57
MF	GO:0005178	integrin bindi	156/18410	2.84E-19	3.72E-17	39
MF	GO:1901681	sulfur compo	267/18410	2.99E-19	3.72E-17	51
MF	GO:0005518	collagen bind	68/18410	5.84E-12	6.04E-10	20
MF	GO:0019838	growth factor	139/18410	9.72E-12	8.63E-10	28

MF	GO:0030020	extracellular	41/18410	7.86E-11	6.10E-09	15
MF	GO:0003779	actin binding	439/18410	3.92E-08	2.46E-06	46
MF	GO:0001968	fibronectin bi	31/18410	3.96E-08	2.46E-06	11
MF	GO:0050840	extracellular	55/18410	6.84E-08	3.86E-06	14
MF	GO:0001664	G protein-cou	288/18410	1.46E-07	7.55E-06	34
MF	GO:0030021	extracellular	22/18410	1.66E-07	7.95E-06	9
MF	GO:0019955	cytokine bind	141/18410	2.06E-07	9.13E-06	22
MF	GO:0048018	receptor ligar	489/18410	3.70E-07	1.53E-05	47
MF	GO:0030546	signaling rece	496/18410	5.57E-07	2.16E-05	47
MF	GO:0008307	structural cor	42/18410	1.29E-06	4.70E-05	11
MF	GO:0004115	3',5'-cyclic-AI	16/18410	2.41E-06	8.31E-05	7
MF	GO:0048407	platelet-deriv	11/18410	2.62E-06	8.58E-05	6
MF	GO:0061134	peptidase reg	230/18410	3.08E-06	9.58E-05	27
MF	GO:0019199	transmembra	79/18410	7.37E-06	0.00021809	14
MF	GO:0004714	transmembra	60/18410	9.09E-06	0.00025668	12
MF	GO:0043394	proteoglycan	36/18410	1.81E-05	0.00048971	9
MF	GO:0030414	peptidase inh	187/18410	2.40E-05	0.00060672	22
MF	GO:0005520	insulin-like gr	29/18410	2.44E-05	0.00060672	8
MF	GO:0017147	Wnt-protein	30/18410	3.20E-05	0.00076521	8
MF	GO:0004114	3',5'-cyclic-nu	23/18410	3.95E-05	0.0009078	7
MF	GO:0097493	structural mo	11/18410	5.85E-05	0.00129799	5
MF	GO:0005516	calmodulin bi	200/18410	6.71E-05	0.00143769	22
MF	GO:0004112	cyclic-nucleot	25/18410	7.17E-05	0.00148392	7
MF	GO:0005179	hormone acti	122/18410	8.31E-05	0.00166409	16
MF	GO:0002020	protease binc	136/18410	9.27E-05	0.00179823	17
MF	GO:0008179	adenylate cyc	12/18410	9.67E-05	0.00182002	5
MF	GO:0061135	endopeptidas	194/18410	0.00012263	0.00218989	21
MF	GO:0004866	endopeptidas	180/18410	0.00012341	0.00218989	20
MF	GO:0042805	actinin bindir	36/18410	0.00013125	0.00226419	8
MF	GO:0031994	insulin-like gr	13/18410	0.0001515	0.00254296	5
MF	GO:0051393	alpha-actinin	28/18410	0.00015744	0.00257304	7
MF	GO:0005109	frizzled bindi	38/18410	0.00019631	0.00312617	8
MF	GO:0005161	platelet-deriv	14/18410	0.0002272	0.00344158	5
MF	GO:0047555	3',5'-cyclic-GI	14/18410	0.0002272	0.00344158	5
MF	GO:0008083	growth factor	162/18410	0.00026459	0.00391253	18
MF	GO:0051371	muscle alpha	15/18410	0.00032858	0.00474101	5
MF	GO:0004867	serine-type e	98/18410	0.00033589	0.00474101	13
MF	GO:0099106	ion channel r	138/18410	0.0003531	0.00487316	16
MF	GO:0017046	peptide horm	52/18410	0.00037888	0.00511534	9
MF	GO:0042562	hormone bin	87/18410	0.0003915	0.00517318	12
MF	GO:0098631	cell adhesion	64/18410	0.00042961	0.0055585	10
MF	GO:0008191	metalloendo	16/18410	0.0004608	0.00575917	5
MF	GO:0050431	transforming	24/18410	0.00046914	0.00575917	6
MF	GO:0051015	actin filamen	214/18410	0.00047294	0.00575917	21

MF	GO:0016247	channel regul	143/18410	0.00052679	0.00629158	16
MF	GO:0008528	G protein-cou	148/18410	0.00076908	0.00901205	16
MF	GO:0005044	scavenger rec	47/18410	0.00089215	0.01007409	8
MF	GO:0015026	coreceptor ac	47/18410	0.00089215	0.01007409	8
MF	GO:0004857	enzyme inhib	390/18410	0.00102635	0.01138239	31
MF	GO:0001653	peptide recep	154/18410	0.00117951	0.0128515	16
MF	GO:0030246	carbohydrate	270/18410	0.00181728	0.01945907	23
MF	GO:0033691	sialic acid bin	22/18410	0.00223378	0.02351351	5
MF	GO:0005246	calcium chan	43/18410	0.00242412	0.02509178	7
MF	GO:0030552	cAMP binding	23/18410	0.00275282	0.02802695	5
MF	GO:0005245	voltage-gatec	45/18410	0.00316566	0.03171033	7
MF	GO:0004222	metalloendo	111/18410	0.00336098	0.0331325	12
MF	GO:0005104	fibroblast gro	25/18410	0.00404357	0.03923854	5
MF	GO:0022843	voltage-gatec	144/18410	0.00428287	0.04092136	14
MF	GO:0030551	cyclic nucleot	37/18410	0.00499926	0.04704251	6
